# The current increase and future perspectives of the microbial pesticides market in agriculture: the Brazilian example

**DOI:** 10.3389/fmicb.2025.1574269

**Published:** 2025-08-11

**Authors:** Matheus Felipe de Lima Andreata, Silas Mian, Galdino Andrade, Adeney de Freitas Bueno, Mauricio Ursi Ventura, José Eduardo Marcondes de Almeida, Eduardo Augusto Fonseca Ivan, Mirela Mosela, Ane Stéfano Simionato, Renata Rodrigues Robaina, Leandro Simões Azeredo Gonçalves

**Affiliations:** ^1^Microbiology Department, Universidade Estadual de Londrina (UEL), Londrina, Brazil; ^2^Agronomy Department, Universidade Estadual de Londrina (UEL), Londrina, Brazil; ^3^BIOINPUT Research Company, Cambé, Brazil; ^4^Embrapa Soja, Londrina, Brazil; ^5^Instituto Biológico de São Paulo, São Paulo, Brazil; ^6^BRANDT Brasil, Cambé, Brazil

**Keywords:** biological control, biofungicides, bioinsecticides, bionematicides, regenerative agriculture

## Abstract

The Brazilian agricultural sector contributes 25% to the national gross domestic product (GDP) and accounts for 49% of the country’s exports, faces significant challenges associated with tropical agriculture. Pests and diseases are major issues that compromise the productivity of various crops. In response, microbial pesticides have increasingly been incorporated as a component of integrated pest and disease management (IPM and IDM, respectively). This study presents a comprehensive analysis of the Brazilian biopesticide market, focusing on bioinsecticides, bionematicides, and biofungicides. Microbial agents, such as *Bacillus* spp., *Trichoderma* spp., and *Beauveria* spp., play a important role in IPM and IDM strategies, acting through multiple biocontrol mechanisms. The biopesticide market in Brazil has grown rapidly, driven by increased adoption by farmers and recent regulatory advances that have facilitated these products’ registration and commercialization process. Projections indicate that this sector will continue to grow in the coming years, supported by research innovations, consolidating biopesticides as key elements in Brazil’s transition to more sustainable agriculture. This review explores the challenges, opportunities, and future trends of microbial pesticides in Brazilian agriculture, highlighting their potential in increasing crop resilience and productivity while reducing the environmental impact associated with conventional pesticides.

## 1 Introduction

Sustainable intensification of agriculture is crucial for global food security, and its population is expected to reach 9.6 billion by 2050 (Tripathi et al., 2019). Brazil is highlighted as a major agricultural player among the most important countries where intensive agriculture is required to advancing this important agenda. Therefore, agribusiness is an important sector of the Brazilian economy, accounting for ¼ of the country’s gross domestic product (GDP) and 49.3% (US$165.55 billion) of exports in 2023. Approximately 40.4% of the foreign revenue in this sector comes from soybeans and their byproducts (grain, meal, and oil). In addition to being the world’s largest exporter and producer of soybeans, Brazil is also considered a leading global producer of other economically important crops, such as sugarcane, corn, coffee, cotton, beans, oranges, and bananas.^1^ Despite the positive agricultural outlook for these crops, significant concerns have arisen due to biotic and abiotic stress, leading to considerable losses in yield in terms of amount and quality.

Global productivity losses caused by pests and crop diseases threaten economies and food security, particularly in developing countries (Ristaino et al., 2021; Singh et al., 2023). It is estimated that, on average, these losses are 21.5% (10.1–28.1%) for wheat, 30.3% (24.6–40.9%) for rice, 22.5% (19.5–41.1%) for maize, 17.2% (8.1–21%) for potatoes, and 21.4% (11–32.4%) for soybeans (Savary et al., 2019). However, the most significant losses are observed in regions with food deficits, rapid population growth, and emerging and reemerging pests and diseases (Savary et al., 2019). The estimated annual cost to the global economy due to losses from pests and diseases exceeds US$220 billion (FAO, 2022).

Over the past few decades, chemical control has become important in mitigating crop losses caused by pests and diseases (Ansari et al., 2014). Despite advancements in alternative strategies, crop protection remains the predominant approach among farmers (Serrão et al., 2022). However, the heavy reliance on traditional chemicals in agriculture poses risks to human health and the environment (Gagic et al., 2021; Jacquet et al., 2022). In a systematic review, Lopes-Ferreira et al. (2022) reported that occupational exposure to pesticides in Brazil is associated with several adverse health effects in farm workers, including hematological, endocrine, neurological disorders, and cancer. This situation is intensified by permissive regulations that allow the use of substances banned in other countries and reclassify highly toxic compounds into less restrictive categories (Pereira et al., 2022; Souza et al., 2023).

In addition to human health concerns, chemical pesticides are also associated with the reduction of natural biological control agentes (Torres and Bueno, 2018) and pollinators (dos Santos et al., 2018), while driving the evolution of pest and disease resistance (Serrão et al., 2022; Gong et al., 2023). Consequently, reducing the use of synthetic chemicals in agriculture to mitigate their adverse effects and pursuing more sustainable management practices (Maciel and de Freitas Bueno, 2023) have become global goals and government policies in various countries worldwide (Lee et al., 2019).

Additionally, managing plant pests and controlling diseases are significant components of agricultural production costs. Global spending on chemical control in agriculture is expected to increase from US$50.62 billion in 2017 to US$68.82 billion by the end of 2025 (Gagic et al., 2021). In this context, adopting integrated control measures such as integrated pest management (IPM) and integrated disease management (IDM) is considered an important strategy for enhancing control effectiveness (Dara, 2019; Abbas et al., 2022; Richard et al., 2022). IPM and IDM are sustainable decision-based approaches that integrate cultural, biological, behavioral, genetic, and chemical tools to identify, manage, and mitigate pest and disease risks. Biological control has emerged as a key strategy because of its effectiveness, minimal environmental impact, and direct benefits to plants, including enhanced growth and the activation of both local and systemic resistance (Collinge et al., 2022). Moreover, the development of commercial biological products is on average 75 times more cost-effective than that of synthetic chemical pesticides (Ram et al., 2018).

**Figure 1:**
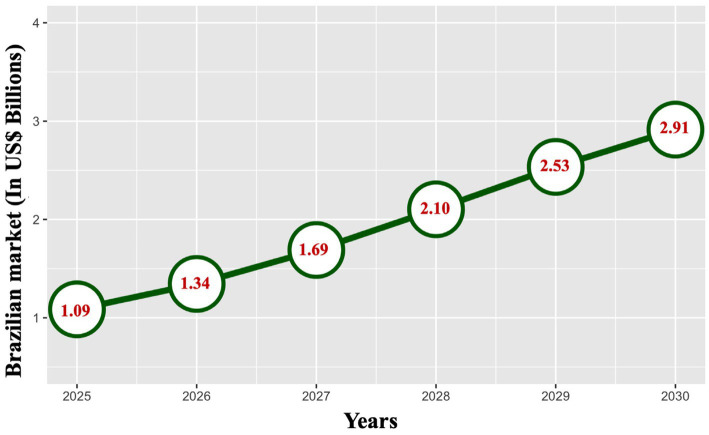
Projection of the Brazilian market for biologicals in pest control. (Exchange rate: US$ 1.00 = R$ 5.80.) Source: Adapted from Borsari and Vieira (2022) copyright Embrapa, 2022.

Therefore, the use of biological control measures has increased worldwide. More than 15% annual growth in the global biological control market had been recorded since reaching a value of US$3.3 billion in 2017 (van Lenteren et al., 2021) and approximately US$14 billion in 2023.^2^ Projections indicate accelerated expansion of this market, which could reach US$49 billion by 2032. Biological control products in the market increased by 71.6% in a few years in Brazil alone. It went from 225 products in 2019 to 386 at the end of 2021 (De Bueno et al., 2023b). Data from Blink Consultancy^3^ indicate that Brazil’s biopesticide market reached US$690 million in the 2023/24 season, accounting for approximately 6% of the total agricultural pesticide market in the country. Among the product categories, bioinsecticides constituted 42.5% of the biopesticide sector, followed by bionematicides (30%) and biofungicides (27.5%). Market projections suggest sustained expansion in these segments, with the sector potentially doubling in size within the next 2–4 years, surpassing US$1.69 billion annually by 2027 and reaching an estimated US$2.91 billion by 2030 (Figure 1) (Borsari and Vieira, 2022). By the end of 2024, the Ministry of Agriculture, Livestock, and Supply (MAPA) had registered 309 products as bioinsecticides, 119 as biofungicides, and 96 as bionematicides,^4^ illustrating the importance of Brazilian agriculture as a case study for adoption of biological control.

Consequently, we analyzed Brazil’s microbial pesticide market in this study by examining the status of product registrations in MAPA and recent trends. It also projects the sector’s trajectory within Brazilian agriculture, identifying the key drivers, challenges, and opportunities that may influence its development in the coming years.

## 2 Microbial biopesticides—mode of action

Biopesticides derived from microorganisms such as bacteria, fungi, viruses, and entomopathogenic nematodes are increasingly recognized for their effectiveness in controlling pests and diseases (Fenibo et al., 2021; Marrone, 2024). Biological control agents (BCAs) play an important role in both IPM (De Bueno et al., 2023a) and IDM (Ayilara et al., 2023) and can be applied either alone or in combination with synthetic pesticides (Maciel et al., 2024). This approach enhances control efficacy and reduces environmental risks and farmers’ and consumers’ exposure to synthetic pesticides. Moreover, microbial biopesticides help manage pest and pathogen resistance to chemical pesticides, a major issue resulting from the prolonged use of synthetic products (Ayilara et al., 2023; Koul, 2023).

**Figure 2:**
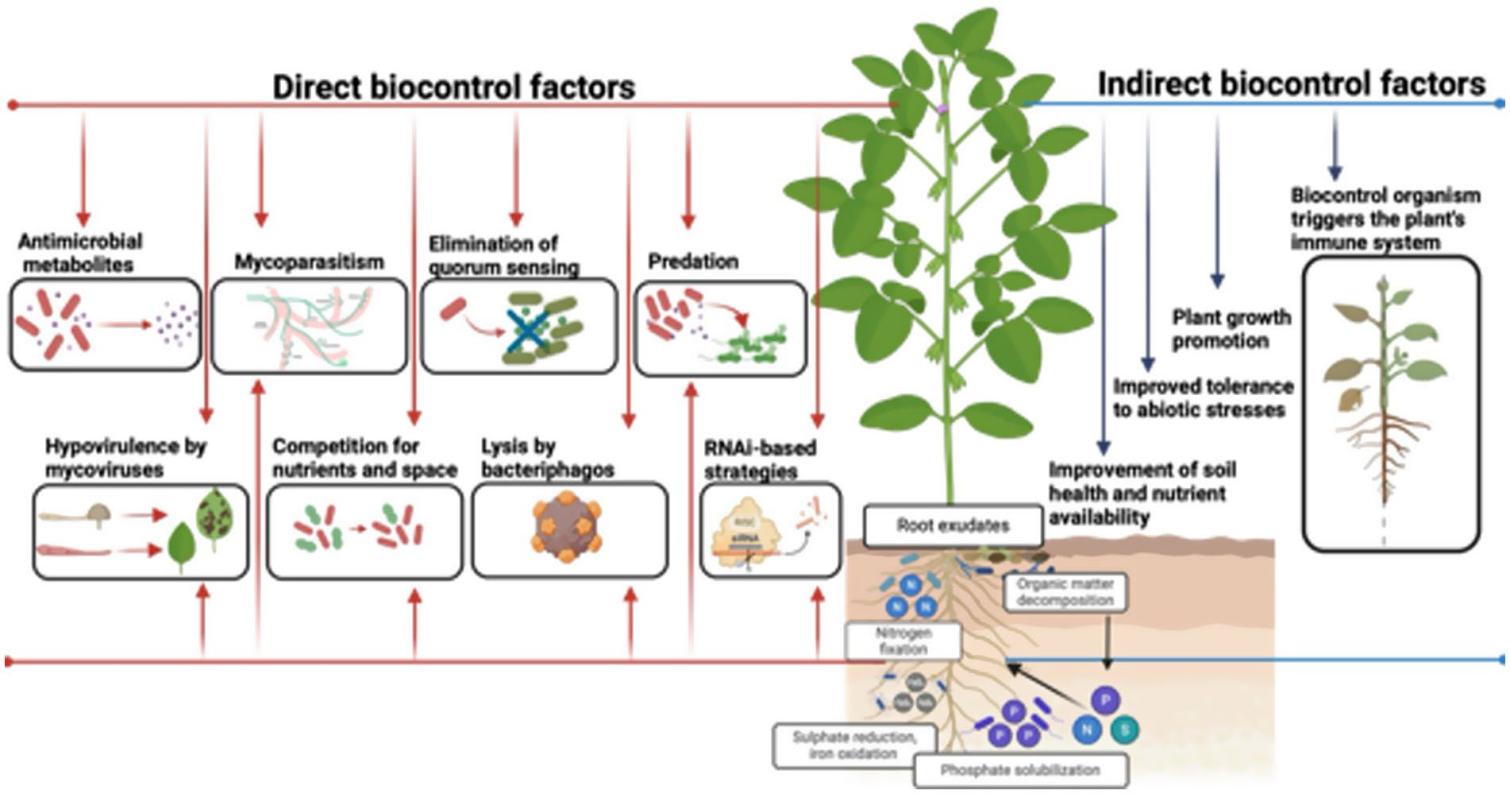
Mechanisms of action of microbial biopesticides in disease and pest management. This figure illustrates the complex interactions and pathways through which microbial biopesticides affect plants, including antagonism, competition, induced systemic resistance, and the key mechanism of direct pathogen inhibition. Additionally, it highlights the role of root exudates in shaping the rhizosphere microbiome and enhancing nutrient cycling processes, such as nitrogen fixation, phosphate solubilization, and organic matter decomposition. Source: Figure created using BioRender.com.

BCAs can control pests and diseases, either directly or indirectly (Figure 2). These microorganisms directly produce secondary metabolites, including specific toxins that disrupt essential cellular processes such as protein synthesis and cell membrane integrity (do Nascimento et al., 2022; Marrone, 2024). Additionally, many BCAs produce lytic enzymes, such as chitinases and cellulases, which degrade the essential structural components of various pests and pathogens, leading to the breakdown of their physical defenses (Poria et al., 2021; Boro et al., 2022; Pandit et al., 2022). Mycoparasitism is another strategy by which beneficial fungi directly parasitize pathogens and destroy their cellular structures (Zaki et al., 2020; Dou et al., 2022). Disruption of quorum sensing, which prevents chemical communication between pathogenic cells, is important in preventing these cells from coordinating virulent attacks on plants (Maddela et al., 2020). Another BCA mode of action is microbial predation, in which predatory microbial species capture, ingest, and digest pathogenic microorganisms or pests. This process is a natural biological mechanism by which predatory microorganisms, such as certain bacteria, fungi, or protozoa, attack and consume pathogenic microorganisms or pests (Köhl et al., 2019; Vero et al., 2023). Other mechanisms that reduce pathogen virulence and competition for space and nutrients, such as hypovirulence induced by mycoviruses, are essential for limiting the growth of pathogenic microorganisms (Vero et al., 2023).

RNA interference (RNAi) has emerged as a promising and specific strategy for managing pests and pathogens in agriculture. By enabling the silencing of essential genes in target organisms through the application or in planta expression of double-stranded RNAs (dsRNAs), RNAi leads to gene knockdown that can impair development, reproduction, or even cause mortality (Adeyinka et al., 2020; Rodríguez Melo et al., 2023).

As an indirect defense mechanism in plants, BCAs can induce systemic resistance and enhance resilience to biotic and abiotic stress (Lahlali et al., 2022). These mechanisms include the activation of complex signaling networks involving phytohormones, such as salicylic acid, jasmonic acid, and ethylene, which are essential for defense against pathogens and pests, as well as for mediating plant-microorganism interactions (Elnahal et al., 2022; El-Saadony et al., 2022; Abdelaziz et al., 2023). BCAs such as *Bacillus* spp. and *Trichoderma* spp. induce systemic acquired resistance (SAR), priming plants with a rapid and robust immune response against a broad range of pathogens (Choudhary and Johri, 2009; Salwan et al., 2022; Rabari et al., 2023). Additionally, BCAs modulate the expression of defense-related genes, including those encoding enzymes such as chitinases and pathogenesis-related proteins (PR proteins), and facilitate the accumulation of biochemical compounds that restrict pathogen spread. Moreover, BCAs with ACC deaminase activity prevent ethylene-induced premature senescence, while promoting plant growth by producing growth regulators and improving nutrient availability (Lahlali et al., 2022). These complex and interconnected mechanisms demonstrate the effectiveness of microbial biopesticides in inducing broad-spectrum resistance and underscore their importance in regenerative agriculture.

## 3 Biopesticides market in Brazil

**Figure 3:**
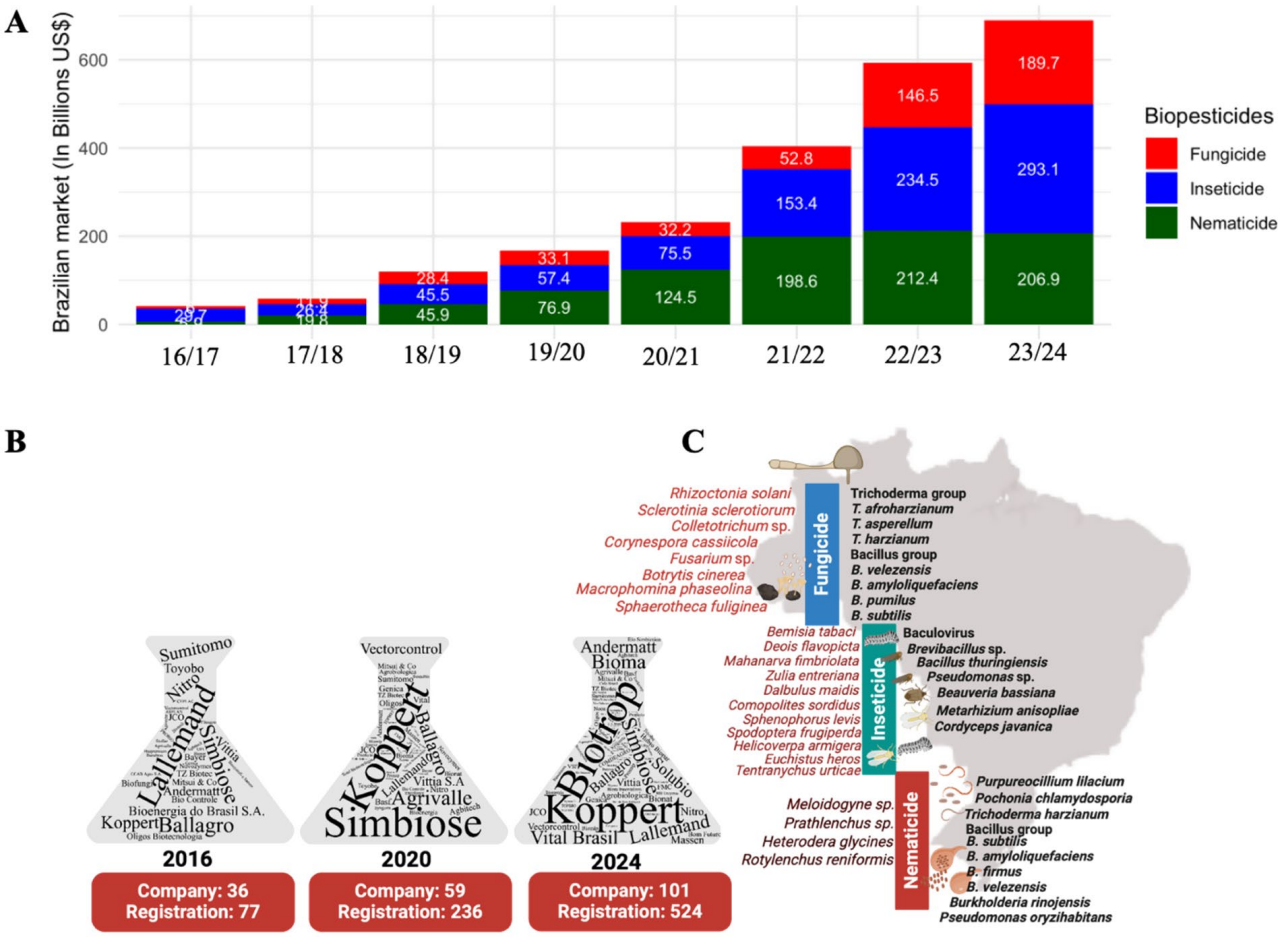
Market dynamics, key targets, and microbial species applied to Brazil’s biocontrol of diseases and pests. **(A)** Growth of the biopesticide market in Brazil over the years, highlighting the number of microbial insecticide, nematicide, and fungicide registrations. **(B)** The distribution of companies and the number of microbial biopesticide registrations underscore the sector’s diversity and representativeness and showcase the industry’s breadth and depth. **(C)** Major target diseases and pests, and the predominant microbial species used as biopesticides. (Exchange rate: US$1.00 = R$5.80.).

The biopesticide market has grown remarkably in Brazil in recent years, following the global trend. As previously mentioned, the sector’s revenue, below US$58 million in 2017, reached US$690 million in the 2023/24 season, reflecting a compound annual growth rate (CAGR) of 49.38% (Figure 3A). When analyzed by segment, the CAGRs were 38.72, 66.38, and 63.65% for insecticides, nematicides, and fungicides, respectively, demonstrating the expansion and increasing adoption of these technologies in Brazilian agribusiness.

Data from MAPA (See text footnote 4) indicate that Brazil had 77 registered biocontrol products from 36 companies in 2016 (Figure 3B). By 2020, this number more than tripled, reaching 236 registrations from 59 companies. As of December 2024, the total number of products rose to 515 across 101 companies. Although many companies focus on specific biocontrol categories, portfolio diversification trends exist. Despite the market’s fragmentation and predominance of small national companies, the sector is steadily maturing. Currently, Brazil’s biocontrol market provides a diverse array of products that target major agricultural pests and diseases (Figure 3C).

Economic, environmental, regulatory, and technological factors drive the expansion of Brazil’s biopesticide market. Establishing a legal framework for bioinputs has played a role in streamlining the registration and commercialization of biological products, fostering an environment conducive to innovation and market growth. At the same time, support from government policies to support sustainable agriculture, together with research advances led by public and private institutions, has been fundamental to the consolidation of this technology. The Brazilian industry has also undergone professionalization to enhance the quality and efficacy of biocontrol agents. The entry of new companies and the diversification of product portfolios further reflect the sector’s growing maturity and resilience.

### 3.1 Brazilian legislation

Brazil has improved its regulatory framework for biological products in alignment with expanding its biopesticide market. The legal landscape governing bioinputs has undergone transformations marked by key legislative milestones.

- Pesticide Law (1989): The first legal framework regulating agricultural inputs, this law broadly classified “products and agents of physical, chemical, or biological processes” but lacked precise definitions for BCAs. This ambiguity posed regulatory challenges for bioinputs.
- Decree No. 4074/2002: An advancement that formally defined BCAs and established precise registration requirements, including taxonomic classification, labeling, concentration, stability, and mode of action. In the same year, Agência Nacional de Vigilância Sanitária (ANVISA) introduced toxicity assessment criteria for vertebrates requiring acute, chronic, mutagenic, carcinogenic, and reproductive toxicity evaluations.
- Joint Normative Instructions (2005–2006, updated in 2014): As the sector grew, the regulatory framework expanded to include microbiological, biochemical, semiochemical, and macrobiological products through joint instructions issued by MAPA, IBAMA, and ANVISA. Under this framework, ANVISA assesses human toxicity; IBAMA evaluates environmental impacts, including risks to non-target species; and MAPA determines field efficacy.
- Joint Ordinance SDA/MAPA, IBAMA, and ANVISA No. 1/2023: Published on April 4, 2023, this ordinance replaced Joint Normative Instruction No. 3/2006, introducing updated registration procedures for microbiological products used in pest and disease control. It also redefines microbiological products to include both active microorganisms and their metabolites.
- Law No. 14785/2023: Repeated the 1989 Pesticide Law, removing outdated classifications and setting the foundation for a more specialized regulatory framework for bioinputs.
- Law No. 15.070/2024: Established a dedicated regulatory framework for bioinputs, removing biological products from the scope of pesticide and fertilizer regulations. Key provisions include a single registration system for multifunctional products, simplified approval procedures for similar bioinput products, and authorization for active ingredient registration for on-farm production. The law exempts on-farm bioinput production from registration, provided that commercial sales remain prohibited. This practice must utilize strains sourced from accredited germplasm banks or registered products approved for this purpose. In addition, the law permits the production and transport of bioinputs among cooperative members and associations, ensuring compliance with good manufacturing practices and supervision by qualified technical professionals.

### 3.2 Microbiological insecticides

**Figure 4:**
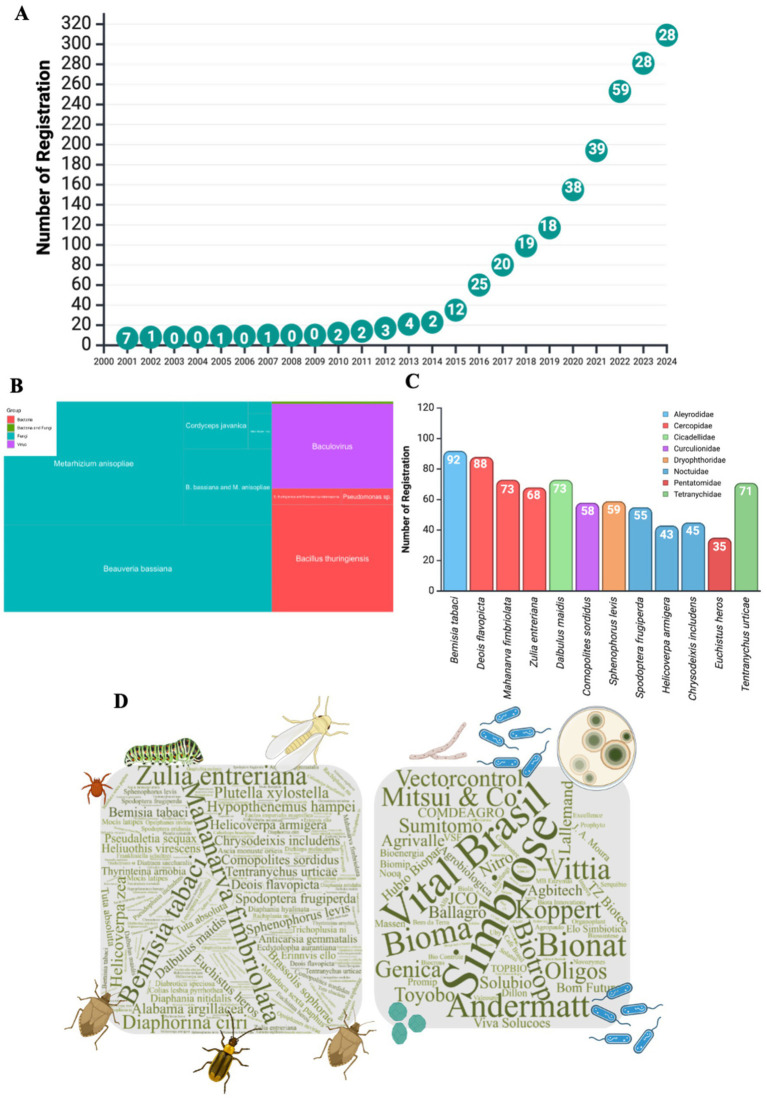
Registrations of microbial insecticide in Brazil: Market trends, key pathogens, and microbial species. **(A)** Annual trend in the number of microbial insecticide registrations in Brazil, reflecting the growing adoption of biocontrol agents. **(B)** The treemap visualizes the predominant microbial species utilized for pest biocontrol, categorized by organism type (bacteria, fungi, or viruses). **(C)** Registration counts for the most targeted pests, showing the relative focus on different pest species. **(D)** Word cloud illustrating the pests with the highest number of microbiological insecticide registrations and the leading companies involved in their registration in Brazil, highlighting the industry scenario.

The number of registered microbial insecticides in Brazil has grown exponentially since 2014 (Figure 4A; Supplementary Table S1). The number of registrations remained extremely low between 2001 and 2013, with fewer than five new registrations annually. However, starting in 2014, there was a significant acceleration with a steady increase in registrations and target pests. This growth can be attributed to regulatory, technological, and marketing factors. The introduction of *Helicoverpa armigera* in Brazil and the occurrence of major outbreaks of this pest in 2013, resulting from unbalanced agriculture that relied exclusively and excessively on chemical control at the time, also reignited interest in IPM and the adoption of biological control by Brazilian farmers (de Freitas Bueno and Sosa-Gómez, 2014).

The introduction of the reference strains *Beauveria bassiana* IBCB 66 and *Metarhizium anisopliae* IBCB 425 by the MAPA in 2007 streamlined the biopesticide registration process. By establishing reliable and widely accepted standards, this measure significantly reduced regulatory complexity and production costs, particularly benefiting small and medium-sized companies. Consequently, it has facilitated market entry for new companies and accelerated the commercialization of biopesticides (Mascarin et al., 2024; McGuire and Northfield, 2020; Mesquita et al., 2023). Currently, 80 and 96% of *B. bassiana* and *M. anisopliae* registrations in Brazil, respectively, are linked to these reference strains. Beyond regulatory advancements, capacity-building initiatives have become instrumental in strengthening the sector. Research institutions such as the Biological Institute, Brazilian Agricultural Research Corporation (EMBRAPA), and public universities have developed specialized training programs focused on the quality control and production of entomopathogenic fungi via solid fermentation. These programs have ensured that companies adopt rigorous production standards, enhance the quality and efficacy of biopesticides, and drive broader adoption in the field.

Research conducted by the “Luiz de Queiroz” College of Agriculture (ESALQ-USP) has been fundamental in driving innovation and developing microbial insecticides in Brazil. This institution is recognized for its studies on entomopathogenic fungi, such as *B. bassiana*, *M. anisopliae*, and *Cordyceps javanica* (formerly known as *Isaria fumosorosea*). Notably, most *C. javanica* registrations in Brazil were derived from strains developed by ESALQ, including ESALQ-1296, ESALQ-3422, and ESALQ-4778. In addition, SparcBio,^6^ an innovation platform that connects academic research to the production sector, has played an important role in accelerating the development of new biological products. This platform aims to facilitate technology transfer, allowing scientific advances generated in academia to be quickly translated into commercial applications, thereby significantly strengthening Brazil’s biocontrol market.

The entomopathogenic fungi *B. bassiana*, *M. anisopliae*, and *C. javanica* accounted for 68% of the microbial insecticide registrations in Brazil (Figure 4B), with *B. bassiana* and *M. anisopliae* being the most prominent with 91 and 83 registrations, respectively, in addition to 22 products that combined both species. *B. bassiana* is widely used to control pests such as the whitefly (*Bemisia tabaci*), sugarcane weevil (*Sphenophorus levis*), brown stink bug (*Euschistus heros*), and pasture and corn spittlebugs (*Deois flavopicta* and *Dalbulus maidis*). *M. anisopliae* is especially effective in controlling spittlebugs such as *Mahanarva fimbriolata*, *Deois flavopicta*, and *Zulia entreriana*. Additionally, two products based on *M. rileyi* targeted fall armyworms (*Spodoptera frugiperda*) (Mesquita et al., 2023).

Despite the continued growth of the market for entomopathogenic fungi, most commercially available products are based on wettable powder (WP) formulations (71%). The primary production technique for these fungi involves solid-state fermentation on rice substrates to achieve high yields of aerial conidia. However, little emphasis has been placed on developing formulations that extend the shelf life and improve pathogenicity. There is a widespread perception that companies lack investment and interest in formulation research to enhance the efficacy of these bioinsecticides, despite the importance of such improvements being widely highlighted in scientific literature (Mwamburi, 2016; Biryol et al., 2021; Meirelles et al., 2023; Mesquita et al., 2023).

*Bacillus thuringiensis* (*Bt*) has become a cornerstone of biological pest control in Brazilian agriculture, particularly following the widespread outbreak of *Helicoverpa armigera*, which caused severe damage to multiple crops, including maize, soybean, cotton (Czepak et al., 2013). Early attempts to manage this pest using conventional insecticides with diverse modes of action have proven ineffective. As an emergency response, Brazil imported baculovirus-based biopesticides from countries such as Australia and, alongside *Bt*, secured expedited registration with the MAPA for pest control (de Freitas Bueno and Sosa-Gómez, 2014). This has facilitated the rapid deployment of biological control programs, in which the combined use of baculovirus and *Bt* demonstrated high efficacy in suppressing *H. armigera*, driving significant growth in the Brazilian biopesticide market (Polanczyk et al., 2017; do Nascimento et al., 2022).

*Bt*-based bioinsecticides account for 17% of microbial insecticide registrations in Brazil. They are primarily used against key agricultural pests, including *Alabama argillacea*, *Chrysodeixis includens*, *Spodoptera frugiperda*, *Ecdytolopha aurantiana*, *Anticarsia gemmatalis*, and *H. armigera*. Among the registered products, the HD-1 strain dominated, representing 51% of approvals, with concentrated suspensions (SC) and WP comprising 61 and 22% of the formulations, respectively.

In response to evolving pest pressures, new bioinsecticide registrations have introduced formulations that combine *Bt* with *Brevibacillus laterosporus*, broadening their spectrum of action and enhancing their appeal to farmers facing simultaneous infestations. This combination offers a dual mode of action: while *Bt* produces insecticidal toxins, *B. laterosporus* actively colonizes the pest’s digestive tract, proliferates, and induces septicemia, further increasing biocontrol efficiency (Smirnova et al., 2023).

Baculoviruses are also recognized as important biocontrol agents, especially for managing pests such as *S. frugiperda*, *A. gemmatalis*, and *H. armigera*, representing 6.7% of the microbial insecticide registrations in Brazil. Their integration into IPM programs has contributed to a reduction in reliance on chemical insecticides. One of the most notable advantages of baculoviruses is their high host specificity, which minimizes the risk to non-target organisms, including pollinators and natural enemies, thereby preserving the ecological balance of agroecosystems.

Another BCA that was registered in 2022 was based on the genus *Pseudomonas*, specifically *P. fluorescens* and *P. chlororaphis*, for the biocontrol of 12 pests (*Bemisia tabaci* biotype B, *Dalbulus maidis*, *Euschistus heros, Diaphorina citri, Aphis gossypii, Dichelops melacanthus*, *Leucoptera coffeella*, *Tetranychus urticae*, *Caliothrips brasiliensis*, and *Frankliniella schultzei*). The mode of action of *Pseudomonas* bacteria in pest biocontrol involves multiple pathogenic mechanisms that enable pest infection and colonization. The success of an infection depends on various virulence factors of *Pseudomonas* spp., such as toxin production, biofilm formation, bacterial motility, and gene regulation. The pathogenic effects of these bacteria on insects include physiological alterations, disruption of feeding behavior and developmental processes, physical deformities, and sepsis, which often results in the death of the infected insects (Teoh et al., 2021).

Figure 4C shows the pests with the highest number of registered products. Eighty-three companies registered microbial insecticides with the MAPA, allowing for the commercialization of these products. Simbiose, Koppert, and Biotrop had the highest number of registrations, with 20, 16, and 16, respectively (Figure 4D).

### 3.3 Microbial nematicides

**Figure 5:**
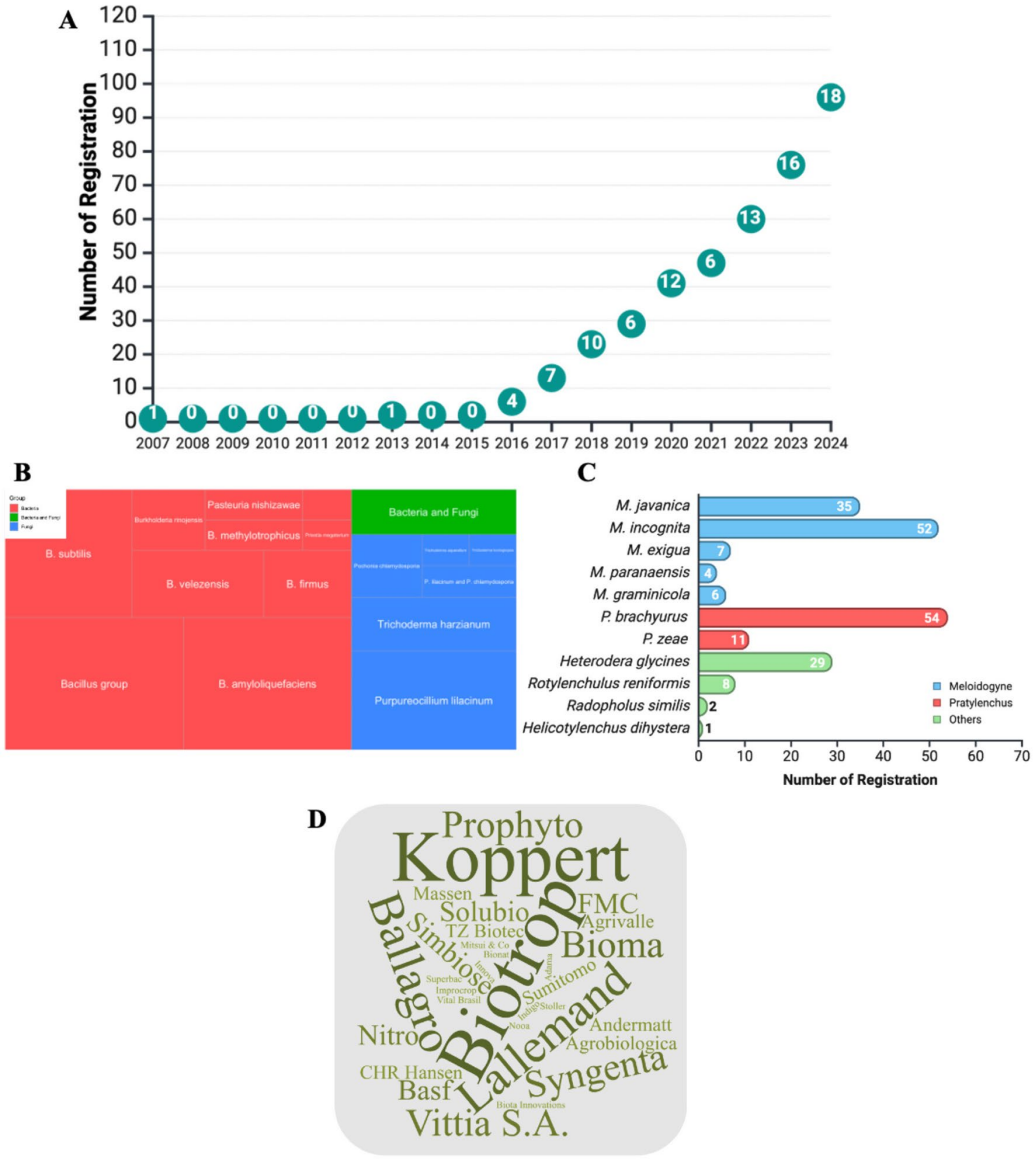
Registrations of microbial nematicides in Brazil: Market trends, key pathogens, and microbial species. **(A)** Annual trends in the registration of microbial nematicides in Brazil, demonstrating the increasing adoption of biocontrol agents for nematode management. **(B)** Treemap illustrating the dominant microbial species used for pathogen biocontrol, categorized by organism type (bacteria, fungi, or a combination of both). **(C)** Number of registrations corresponding to the most frequently targeted nematode species, providing insight into the focus on specific disease-causing species. **(D)** Word cloud showcasing the leading companies involved in registering microbial nematicides in Brazil, highlighting the competitive landscape within the industry.

The registration of nematicides in Brazil has grown significantly over the past few years (Figure 5A; Supplementary Table S2). The number of registrations remained low between 2007 and 2015, reflecting an initially slow adoption of these products with only two registrations (Trichodermil SC and Nemat). However, an upward trend was observed from 2016 onward, with 96 registrations by December 2024, and an average growth of 10 new registrations per year. This increase can be attributed to several interrelated factors that drive farmers to adopt these products. One of the main factors is the devastating effects of plant-parasitic nematodes on Brazilian agricultural areas, causing an estimated loss of US$11.7 billion across various crops. Losses due to nematode infestation reach US$5 billion in soybeans, which is equivalent to losing an entire crop every ten years (Machado, 2022).

Another factor contributing to this market’s growth is the widespread presence of plant-parasitic nematodes in agricultural regions. A recent survey by Syngenta, Agroconsult, and the Brazilian Society of Nematology revealed that 90% of 21,661 samples collected across Brazil contained plant-parasitic nematodes (Syngenta, 2022). The most prevalent nematode genera in Brazil are *Meloidogyne*, *Pratylenchus*, *Rotylenchus*, and *Heterodera*. These genera cause significant damage to key crops such as soybeans, corn, cotton, and sugarcane, leading to substantial reductions in agricultural productivity and a negative economic impact.

The effectiveness of microbial nematicides in controlling plant-parasitic nematodes and promoting plant growth has been demonstrated in the field, increasing farmers’ confidence in, and adoption of, these products (Machado, 2022). Consequently, the market for microbial nematicides will surpass that for chemical nematicides, representing 55, 94, and 100% of the nematicides sold for sugarcane, soybeans, and corn, respectively, in the 2021/2022 season. Projections from Blink Consultancy indicate that this market will continue to grow, with estimated increases of 87, 48, and 86% for the same crops by the 2026/2027 season, attracting the interest of both small and large companies and expanding the range of products available in the market.

In Brazil, the genus *Bacillus* sp. is present in over 60% of commercial microbial nematicide products, either in the consortia of strains from the same or different *Bacillus* species or as single strains of *B. amyloliquefaciens*, *B. velezensis*, *B. subtilis*, and *B. firmus* (Figure 5B). This genus uses various mechanisms to reduce nematode populations, such as (i) regulating nematode behavior by interfering with host recognition, (ii) competing for nutrients, (iii) promoting plant growth, (iv) inducing systemic resistance, and (v) producing metabolites and volatile compounds that inhibit egg hatching, reduce juvenile survival, and directly kill nematodes (Engelbrecht et al., 2018; Aloo et al., 2019; Dimkić et al., 2022; Bhat et al., 2023).

Other bacteria registered for nematode control in Brazil include *Pasteuria nishizawae*, *Pseudomonas oryzihabitans*, and *Burkholderia rinojensis*. The commercial product, Clariva, developed by Syngenta and based on *P. nishizawae*, specifically targets *H. glycines*. In 2023, Indigo registered a new product based on *P. oryzihabitans*, the efficacy of which is associated with producing antimicrobial compounds and cell wall-degrading enzymes, such as chitinases, which directly affect nematode survival and development. Additionally, metabolites of *Burkholderia rinojensis* strain A396, registered by Rizobacter in Brazil and marketed in the United States under the name Majestene® by Marrone Bio Innovations, are noted for their broad-spectrum nematicidal action (Arthurs and Dara, 2019).

Among the fungi registered as microbial nematicides in Brazil, *Purpureocillium lilacinum*, *Trichoderma harzianum*, and *Pochonia chlamydosporia* are notable. These organisms control nematodes through three main mechanisms: direct parasitism, antibiosis, and the induction of plant resistance. *P. lilacinum* and *P. chlamydosporia* primarily target and parasitize nematode eggs. Their hyphae penetrate the egg structure and release enzymes, such as chitinases and proteases, which degrade the protective shell and prevent hatching and juvenile development. Additionally, these fungi produce secondary metabolites with nematicidal properties such as aurovertins and phomalactone, which can kill or inhibit nematode movement (Li et al., 2015; Soares et al., 2023; Ayaz et al., 2024). *T. harzianum* combines both direct and indirect actions for nematode control. In addition to acting as an antagonist by degrading nematode cuticles through lytic enzymes, this fungus also stimulates the natural defenses of plants. By activating the jasmonic acid and salicylic acid signaling pathways, *T. harzianum* induces systemic resistance in plants, making them less susceptible to nematode attacks. Another important mechanism is the production of volatile compounds and secondary metabolites that have direct nematicidal properties or alter the conditions of the rhizosphere, making the environment less favorable for nematode survival (Yao et al., 2023).

The primary targets for microbial nematicide registration in Brazil include *Pratylenchus brachyurus*, *Meloidogyne incognita*, *M. javanica*, and *Heterodera glycines*, with 54, 52, 35, and 29 registered products, respectively (Figure 5C). *Pratylenchus*, particularly *P. brachyurus*, is a migratory endoparasitic nematode that parasitizes many plant species, including soybeans, corn, cotton, rice, and beans. By feeding on the cellular contents of the roots, this nematode causes lesions that lead to necrosis and impaired plant development, resulting in significant yield losses. *P. brachyurus* has emerged as a critical phytosanitary threat in Brazil, especially to soybean production, and can cause crop losses of up to 30%. This impact is amplified in the Cerrado regions, where consecutive planting of soybeans and corn favors the spread of the pathogen. Additionally, *P. brachyurus*’ ability to survive for long periods without a host, coupled with its asexual reproduction, complicates its management (Nomura et al., 2024).

Currently, 31 companies have registered microbial nematicides with the MAPA, authorizing the commercialization of these products in Brazil. Biotrop and Koppert led the market with 14 and 9 registrations, respectively (Figure 5D). However, there are duplications in company registrations that artificially increase the total number of registered products. For example, the *B. subtilis* strain CNPSo 3,602 has six different registrations, and *T. harzianum* strain 1,306 has four registrations. This practice of multiple registrations for the same strain contributes to the inflation of registration numbers in the country, thus masking the actual diversity of products available in the market.

### 3.4 Microbial fungicides

**Figure 6:**
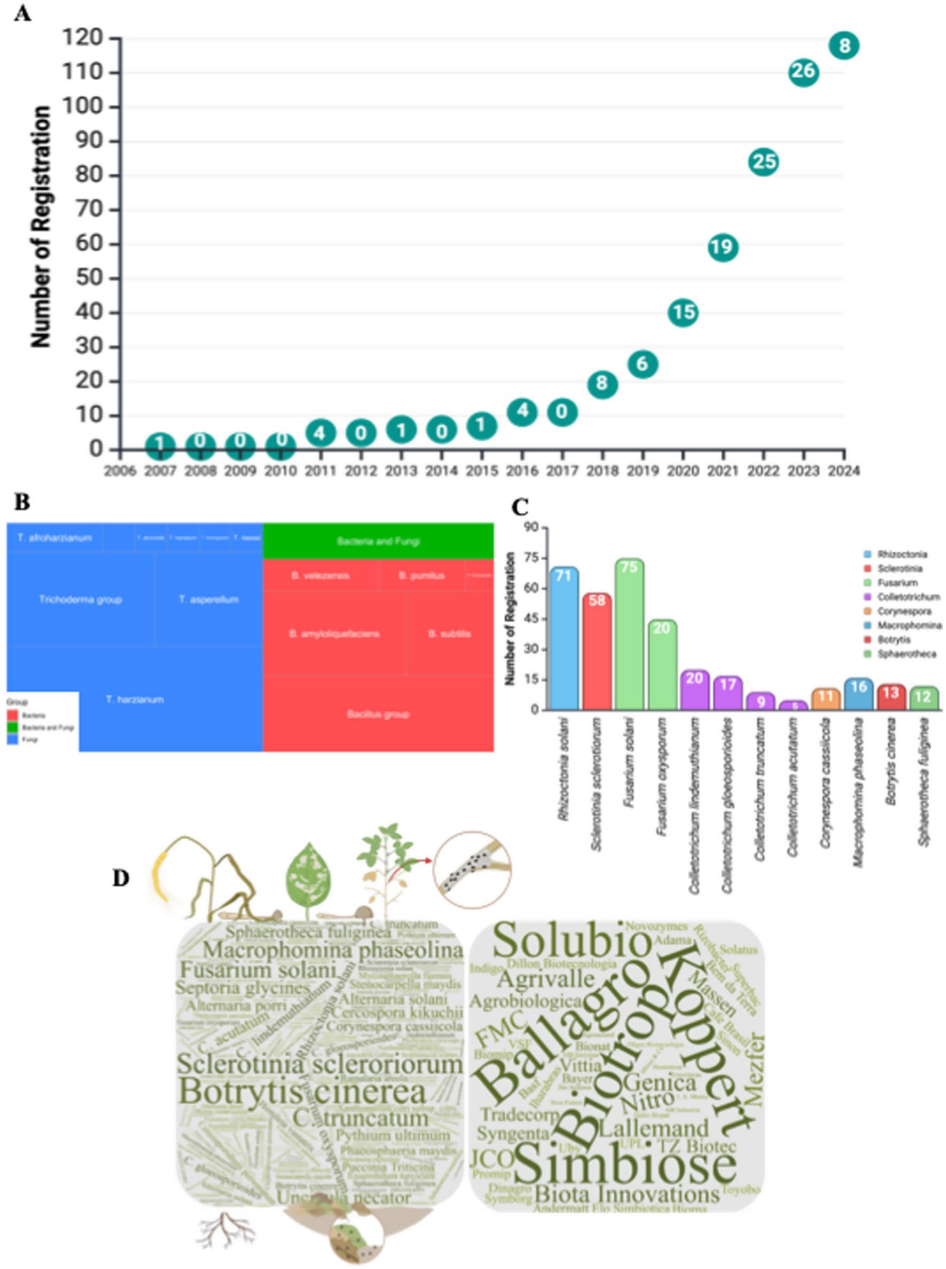
Registrations of microbial fungicides in Brazil: Market trends, key pathogens, and microbial species. **(A)** Annual trends in the registration of microbial fungicides in Brazil, demonstrating the increasing adoption of biocontrol agents for disease management. **(B)** Treemap illustrating the dominant microbial species used for pathogen biocontrol, categorized by organism type (bacteria, fungi, or a combination of both). **(C)** Number of registrations corresponding to the most frequently targeted pathogens, providing insight into the focus on specific disease-causing species. **(D)** Word cloud illustrates the pests with the highest number of microbiological fungicide registrations and Brazil’s leading companies involved in their registration, highlighting the industry scenario.

Following the global trend of increasing use of biological products, the microbial fungicide market has experienced accelerated growth in recent years. Between 2018 and 2024, 107 new products were registered, representing approximately 90% of current microbial fungicides (Figure 6A; Supplementary Table S3). The first microbial fungicide registered in Brazil was *T. harzianum* strain ESALQ 1306 in 2007. Subsequently, *T. asperellum* strains URM-5911 and T211, along with the first bacterial strains, *B. pumilus* QST 2808 and *B. subtilis* QST 713, were registered in 2011. These two microorganisms dominate the fungicide market in Brazil (Figure 6B).

Fungi of the genus *Trichoderma* are widely recognized for their fungicidal properties and roles in the biological control of plant diseases. In Brazil, approximately 57% of the registered biofungicides contain fungi of this genus, with the primary biocontrol targets being: (i) *Fusarium oxysporum* f. sp. *phaseoli*, (ii) *Colletotrichum lindemuthianum*, (iii) *Rhizoctonia solani*, and (iv) *Sclerotinia sclerotiorum*. Trichoderma uses both direct and indirect mechanisms for biocontrol of phytopathogenic fungi. Directly, *Trichoderma* competes with pathogens for space and nutrients, particularly in the rhizosphere, and produces secondary antifungal metabolites such as gliotoxin and 6-pentyl-α-pyrone, which inhibit pathogen growth. Mycoparasitism is another important biocontrol mechanism in which *Trichoderma* secretes lytic enzymes, such as chitinases, glucanases, and proteases, which degrade the cell walls of phytopathogenic fungi, leading to their destruction and death. *Trichoderma* induces systemic resistance in plants by activating defense pathways such as jasmonic acid and ethylene. This “priming” effect strengthens the plant’s immune response, making it more resistant to pathogen attacks by increasing the production of pathogenesis-related proteins and other defense enzymes (Asad, 2022; Dutta et al., 2022; Rodrigues et al., 2023). In Brazil, *T. harzianum* and *T. asperellum* species have the highest number of registered products, followed by *T. viride*, *T. hamatum*, *T. afroharzianum*, and *T. atroviride*.

*Trichoderma*, like entomopathogenic fungi, is traditionally grown on solid substrates, a method known for its simplicity and low cost that facilitates the entry of new companies into the market. This method also promotes the production of high-quality spores with longer shelf lives. However, this method has significant limitations in terms of scalability and automation. In contrast, liquid fermentation offers clear advantages for large-scale production, allowing precise control of variables such as pH, temperature, and aeration and optimizing both fungal growth and the production of secondary metabolites. This results in a more uniform and efficient biomass production over a shorter period than solid substrate fermentation. However, liquid fermentation is more expensive, and the propagules produced have a reduced shelf life, presenting a commercial challenge (Prakash and Basu, 2020). Recent studies have sought to extend the durability of conidia, mainly by adding osmoprotectants to formulations (Sriram et al., 2011; de Rezende et al., 2020; Martinez et al., 2023). Currently, the most common formulations of *Trichoderma*-based products in Brazil are WP and suspension concentrate (SC), which account for approximately 73% of the products on the market. However, in recent years, new formulations have been developed, such as water-dispersible granules (WG), soluble liquid (SL), oil-dispersible (OD), emulsifiable concentrate (EC), emulsifiable gel (GL), and dry suspension (DS), which expand the application options and enhance the use of these biofungicides.

*Bacillus* species available in the Brazilian market can be grouped into four types: *B. amyloliquefaciens*, *B. velezensis*, *B. subtilis*, and *B. pumilus*, which are formulated either individually or in combination with one or more strains. This genus produces a wide range of antimicrobial compounds, including cyclic lipopeptides, such as iturins, fengycins, and surfactins, destabilizing fungal cell membranes and leading to cell death. Additionally, *Bacillus* spp. secrete lytic enzymes, such as chitinases and glucanases, which degrade fungal cell walls and inhibit fungal growth. Indirectly, *Bacillus* induces systemic resistance in plants by activating defense pathways and enhancing immune responses against fungal pathogens. Furthermore, *Bacillus* produces volatile organic compounds such as acetone, inhibiting fungal growth and spore germination, causing morphological changes in fungal cells (Penha et al., 2020; Hirozawa et al., 2023; Serrão et al., 2024). BCAs containing *Bacillus* strains that are registered in Brazil have demonstrated efficacy against 58 fungal pathogens, including soil-borne pathogens, such as *R. solani*, *S. sclerotiorum*, *M. phaseolina*, and *F. solani*. Additionally, these BCAs are effective against foliar fungi and late-season disease-causing pathogens such as *Botrytis cinerea*, *Phakopsora pachyrhizi*, *Sphaerotheca fuliginea*, *Colletotrichum gloeosporioides*, *C. lindemuthianum*, *Corynespora cassiicola*, and *Cercospora kikuchii*.

Historically, the primary targets for BCA registration in Brazil have been soil-borne diseases (Figure 6C). However, these products have grown in adoption for the integrated management of foliar diseases, acting as protective and preventive agents. Some companies now recommend applying biological products during stages V0–V4 for late-season diseases in soybean cultivation to ensure early protection and increase management effectiveness. When combined with chemical fungicides, biological agents offer comprehensive and complementary multisite actions, reducing the risk of pathogen resistance. This comprehensive approach instills confidence in the audience regarding the effectiveness of products. In addition, these products have prolonged residual effects, contributing to more sustainable and efficient long-term management strategies.

Figure 6D shows the central diseases with the most registered products and the companies with the most registrations. According to the MAPA, 58 companies registered microbial fungicides, with Biotrop leading with 13 registrations, followed by Simbiose, Koppert, and Ballagro with 8, 6, and 6 registrations, respectively.

## 4 Future perspectives of biopesticides in Brazil

The outlook for Brazil’s biopesticide market indicates continued growth. The sector expanded by 312% from 2019 to 2024 (5 years), reaching a revenue of US$737 million. Over the next 5 years (from 2024 to 2029), Brazil is expected to lead the global bioinputs market, with the national market projected to reach US$2.71 billion by 2029 and US$3.11 billion by 2030 (Figure 1). This growth is driven by the increasing integration of biotechnological innovations, increasing adoption by farmers, and the need to reduce dependence on chemical pesticides.

Advancing Brazilian legislation and government incentives are fundamental for expanding the biopesticide market. A milestone in this effort was the establishment of the National Bioinput Program in 2020, aiming to accelerate the development and adoption of microbial biopesticides. The key objectives include proposing regulatory frameworks, fostering scientific and technological innovation, expanding access to credit, promoting biofactories, and providing technical training.

**Figure 7:**
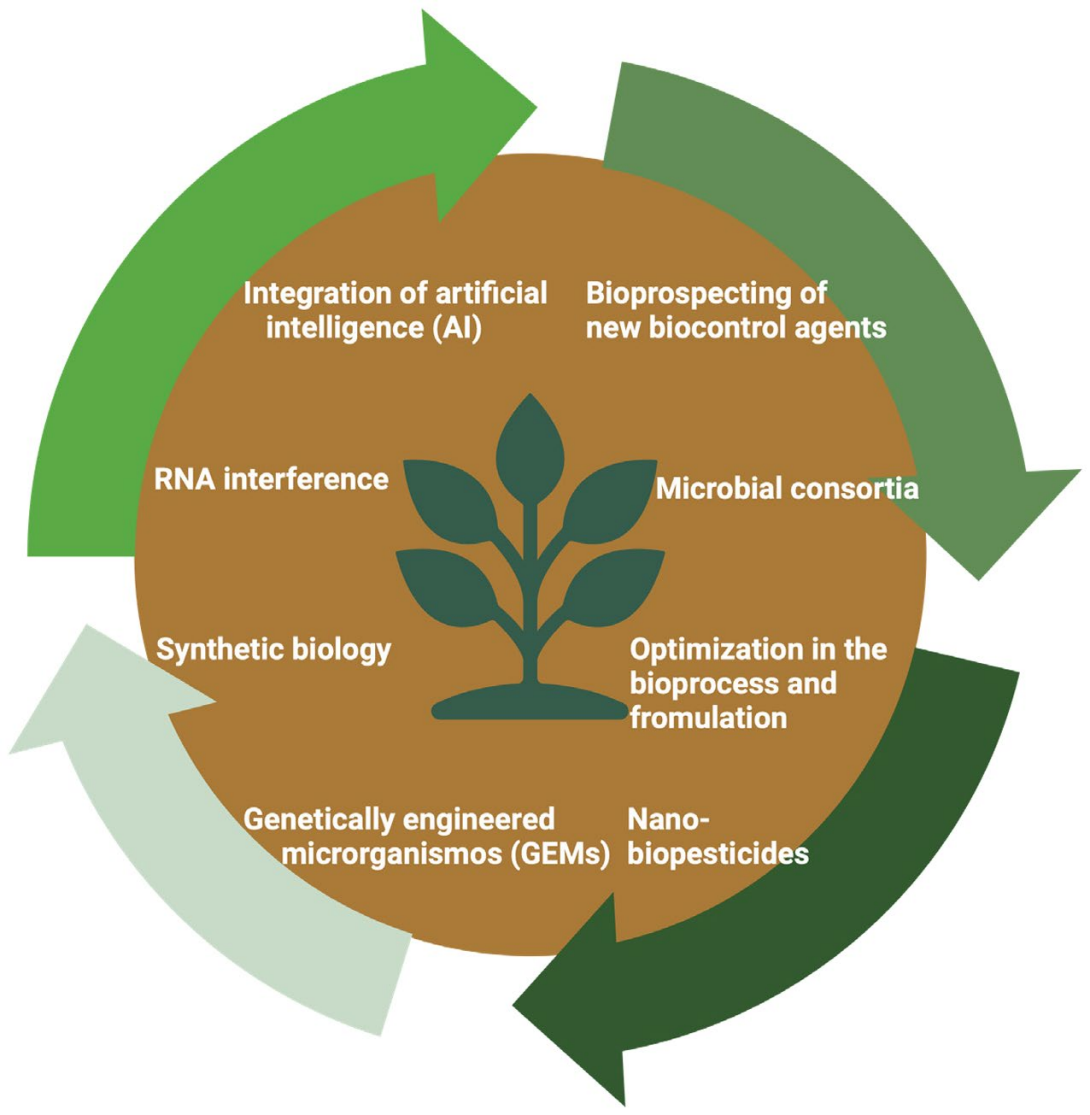
Technologies to drive the development of biopesticides for agriculture.

In the current landscape of technological developments, several research fronts are being explored to boost the use of biopesticides in agriculture (Figure 7). Among these, we highlight the following.

### 4.1 Bioprospecting for new biocontrol agents

The exploration of new strains and microbial species is an important strategy in the development of microbial biopesticides. The discovery of unknown microorganisms, or even those that are already known but underexplored, could reveal BCAs with new applications, thereby increasing the currently available biological control options for pests and diseases (Poveda, 2021). Additionally, advancements in genomic tools, such as metagenomics and next-generation sequencing, have accelerated the identification of bioactive genes and metabolites that can be harnessed for use as biological insecticides, thereby expanding alternatives for pest and disease management (Massart et al., 2015; Zhang et al., 2021). Integrating these tools with the growing understanding of symbiotic and antagonistic interactions in agricultural ecosystems makes it possible to develop more effective biopesticides capable of working synergistically with natural soil microbial communities (Pasin et al., 2021).

### 4.2 Microbial consortia

The development of microbial consortia represents a significant advancement in agricultural biopesticides by providing more effective and resilient pest and disease management strategies. Combining multiple microbial species with complementary functions generates synergistic interactions that enhance biocontrol efficacy and improve plant resilience to biotic and abiotic stresses, including pathogen attack and adverse environmental conditions (Nunes et al., 2024). Microbial consortia optimize key physiological processes such as plant growth promotion and nutrient uptake, while reinforcing plant immunity against pathogens, often outperforming biopesticides based on single strains. In addition to their direct benefits to plant health, these microbial formulations enrich soil biodiversity and foster more stable and sustainable agricultural ecosystems. However, the widespread adoption of microbial consortia requires overcoming critical challenges, such as ensuring species compatibility, maintaining product stability, and mitigating competition with native soil microbiomes (Vishwakarma et al., 2020; Nunes et al., 2024).

### 4.3 Optimization in bioprocessing and formulations

One of bioprocessing’s main focuses is optimizing microbial metabolite production for biocontrol through adjustments to parameters such as nutrient availability and fermentation conditions. These adjustments can significantly improve the yield and stability of the metabolites. Another important aspect is the optimization of liquid fermentation for entomopathogenic fungi, such as *B. bassiana* and *M. anisopliae*, to increase the production of blastospores. However, a principal challenge remains in extending the shelf life of these blastospores, which degrade quickly, thereby limiting their prolonged field application (Mascarin et al., 2024). Microencapsulation has emerged as a promising strategy for protecting microorganisms and their metabolites in soil. This technique encapsulates biological agents in a protective matrix, shielding them from competition with native microbiota and adverse environmental conditions, while allowing for more controlled and gradual release. This enhances their stability, competitiveness, and efficacy in pest and disease control. For foliar applications, the challenge is to protect microorganisms from environmental conditions, such as humidity, UV radiation, and temperature. Research on formulations incorporating protective and stabilizing agents has shown great potential to ensure that microorganisms and their metabolites remain active under different environmental conditions, maximizing their effectiveness in the field.

### 4.4 Nano-biopesticides

Nanotechnology is an important area for the development of biopesticides, with the advent of nanobiopesticides providing advances in terms of stability, controlled delivery, and greater efficacy, allowing the use of smaller doses (Ayilara et al., 2023; Sreevidya et al., 2023). These nanoformulations overcome classical limitations, such as rapid degradation, inconsistent performance, and high production costs, which have historically restricted the widespread adoption of microbial pesticides. By encapsulating microbial agents in nanoparticles, these technologies offer protection against environmental degradation, increase the persistence of the agents in the field, and ensure precise release at the site of action. Additionally, the increased surface area of nanoparticles enhances their interaction with pathogens, boosting bioactivity and maximizing efficacy (Sreevidya et al., 2023).

### 4.5 Genetically modified microorganisms or genetically edited microorganisms

Genetic engineering techniques, such as genomic recombination and gene editing, have been successfully applied to various microorganisms, allowing for precise modification of genes associated with biocontrol activity (Panth et al., 2020; Liu et al., 2022; Puan et al., 2023; Wei et al., 2024). According to Puan et al. (2023), various genetic engineering approaches have been developed to increase the production of antimicrobial metabolites by *Bacillus* spp. Key strategies include modifying promoters and genetic expression systems that enhance the transcription of genes involved in the biosynthesis of antimicrobial peptides (AMPs), such as surfactin, iturin, and fengycin, which are known for their action against agricultural pathogens. Similarly, in *Trichoderma*, genetic engineering promotes the overproduction of lytic enzymes that degrade the cell walls of pathogens and stimulates the synthesis of bioactive compounds with potent antifungal activities (Adnan et al., 2022; Xiao et al., 2023).

Using microbial chassis, such as *Escherichia coli* and *Saccharomyces cerevisiae*, has shown promise for the heterologous production of complex metabolites with high potential as biopesticides. These chasses are robust platforms for large-scale production of agriculturally relevant compounds, opening new frontiers for developing more efficient biological products. However, the use of genetically modified microorganisms (GEMs) raises essential questions regarding their environmental impacts and food safety. Concerns include the potential adverse effects on natural ecosystems, such as unintended interactions with non-target organisms and the spread of modified genes (Shams et al., 2024). In Brazil, the registration and commercialization of microorganisms must undergo risk assessment and obtain prior authorization from the National Technical Biosafety Commission (CTNBio), depending on the assigned risk class. Activities must follow appropriate containment and biosafety protocols, with protection levels ranging from NB-1 to NB-4 according to the level of risk involved. These protocols are designed to minimize the potential escape or accidental release of organisms into the environment.

### 4.6 Synthetic biology

Synthetic biology can revolutionize the development of microbial biopesticides in agriculture, thereby enabling the design and engineering of microbial systems with enhanced functionality and precision (Sargent et al., 2022; Wang et al., 2022; Sreevidya et al., 2023). Synthetic biology facilitates the simultaneous introduction of multiple traits such as enhanced resistance to pathogens, by enabling the redesign of existing biological systems or the creation of new genetic circuits (Sargent et al., 2022). This capability is crucial for developing next-generation microbial consortia and biopesticides that are more tolerant to environmental stresses and more effective in protecting crops (Ke et al., 2021; Basnet et al., 2022). Moreover, the synthetic biology of biopesticides can accelerate the discovery of new metabolic pathways and bioactive compounds, thereby expanding the arsenal of tools available for biocontrol.

### 4.7 RNA interference

RNAi technology utilizes double-stranded RNA (dsRNA) molecules to silence specific genes in target organisms, blocking gene expression at the mRNA level and preventing the production of proteins essential for the development of agricultural pests (Guan et al., 2021; Sharma et al., 2023). This mechanism offers an advantage over conventional chemical pesticides by allowing non-target organisms and beneficial species to remain unharmed. The production of dsRNA in microorganisms, such as *E. coli* and *S. cerevisiae*, has made RNAi-based biopesticides scalable and economically viable (Fletcher et al., 2020; Guan et al., 2021). However, field applications face challenges such as the rapid degradation of dsRNA under adverse environmental conditions, especially when exposed to UV radiation and moisture. Moreover, the efficacy of RNAi varies across insect species and is particularly low in lepidopterans, which exhibit limited dsRNA uptake (Sharma et al., 2023). Recent advancements in RNAi technology, such as the encapsulation of dsRNA in nanoparticles and their conjugation with polymers, have improved the stability and uptake of these molecules, thereby increasing the effectiveness of RNAi in the field (Dalakouras et al., 2024). The U. S. EPA approved the first commercial RNAi-based insecticide, called Calantha, which was developed by GreenLight Biosciences, in 2024. The active ingredient ledprona is a dsRNA molecule that interferes with the expression of the *Snf7* gene in the Colorado potato beetle (*Leptinotarsa decemlineata*), a gene essential for the function of the ESCRT-III complex, which is responsible for cellular protein degradation. Disruption of this gene leads to cellular dysfunction and eventually the death of the insect, offering a specific and effective control for this pest.

### 4.8 Integration of artificial intelligence

Artificial intelligence (AI) offers an innovative and promising approach for developing new microbial pesticides. AI-based tools, such as machine learning, deep learning, predictive modeling, and genetic algorithms, can significantly accelerate bioprospecting by analyzing large volumes of genomic data and identifying microbial species, strains, and genes with high biocontrol potential. This capability enables predicting functional interactions and efficient identification of new bioactive compounds. Additionally, AI can be used to optimize the microbial consortium design and maximize the synergistic interactions between different species or strains, resulting in more effective consortia for biocontrol. AI-driven synthetic biology tools facilitate the creation of genetically modified microorganisms with stability, resilience, and specificity for the control of pests and pathogens (Li et al., 2021; Holzinger et al., 2023).

## 5 Concluding remarks

The biopesticide market in Brazil, favored due to the country’s rich biodiversity and the increasing global demand for reducing agrochemicals in food production, has grown rapidly and will continue to grow. This, supported by innovations and the discovery of new green-control products, makes the country the global leader in the development and adoption of this technology. The replacement of traditional chemical pesticides with bioinputs has transformed agriculture in the country. In a continuous search to increase Brazilian agricultural sustainability, adopting IPM and IDM must be incentivized and expanded nationwide. IPM and IDM promote a more rational use of pesticides and, therefore, a more balanced and favorable agroecosystem for biocontrol. IPM and IDM adoption increase the chances of biocontrol to survive and persist in agroecosystems, thereby promoting sustainable and profitable agriculture. Continuous investments in public and private research and the training of different actors in different production systems of agriculture will facilitate and consolidate the continuously growing biological control market in the country.

## Footnotes

^1^ http://www.ibge.gov.br

^2^ https://www.marketsandmarkets.com

^3^ https://blinkstrategies.com

^4^ https://agrofit.agricultura.gov.br

^6^ https://www.sparcbio.com.br
