# Supplementary material for: The current increase and future perspectives of the microbial pesticides market in agriculture: the Brazilian example
Source: Front Microbiol. 2025 Aug 11;16:1574269. doi: 10.3389/fmicb.2025.1574269 (PMC12375563; doi:10.3389/fmicb.2025.1574269)
Supplement: Supplementary file 1 [file Table_1.DOCX]

**The current increase and future perspectives of the microbial pesticides market in agriculture: The Brazilian example**

Matheus Felipe de Lima Andreata^1^, Silas Mian Alves^2^, Galdino Andrade^1^, Adeney de Freitas Bueno^3^, Mauricio Ursi Ventura^2^, José Eduardo Marcondes de Almeida^4^, Eduardo Augusto Fonseca Ivan^5^, Mirela Mosela^1^, Ane Stéfano Simionato^6^, Renata Rodrigues Robaina^6^, and Leandro Simões Azeredo Gonçalves^2*^

^1^Microbiology Department, Universidade Estadual de Londrina (UEL), Londrina, Paraná, 86057-970, Brazil

^2^Agronomy Department, Universidade Estadual de Londrina (UEL), Londrina, Paraná, 86057-970, Brazil

^3^Embrapa Soja, Londrina, Paraná, 86085-981 Brazil

^4^Instituto Biológico de São Paulo, Campinas, São Paulo, 04014-900, Brazil

^5^BRANDT Brasil, Cambé, Paraná, 86181-570, Brazil

^6^BIOINPUT Research Company, Cambé, Paraná, 86181-570, Brazil

*Corresponding author: [leandrosag@uel.br](mailto:leandrosag@uel.br)

**Table S1.** Overview of registered microbiological insecticide in Brazil.

| **N°** | **Year** | **Company** | **Product name** | **BCAs^1^** | **Targets** |
| --- | --- | --- | --- | --- | --- |
| 1 | 1991 | Sumitomo | Dipel | *Bacillus thuringiensis* var. kurstaki | Ecdytolopha aurantiana, Diatraea saccharalis, Diaphania hyalinata, Alabama argilacea, Ascia monuste orseis, Trichoplusia ni, Heliothis virescens, Anticarsia gemmatalis, Brassolis sophorae, Opsiphanes invirae, Helicoverpa armigera, Pseudaletia sequax, Pseudoplusia includens, Thyrinteina arnobia, Condylorrhiza vestigialis, Tuta absoluta |
| 2 | 1991 | Vectorcontrol | Bac-Control WP | *Bacillus thuringiensis* Berliner | Ecdytolopha aurantiana, Diaphania nitidalis, Diaphania hyalinata, Helicoverpa zea, Alabama argilacea, Ascia monuste orseis, Mocis latipes, Trichoplusia ni, Erinnyis ello, Colias lesbia pyrrhothea, Heliothis virescens, Anticarsia gemmatalis, Brassolis sophorae, Helicoverpa armigera, Eacles imperialis magnifica, Spodoptera frugiperda, Thyrinteina arnobia, Manduca sexta paphus, Plutella xylostella |
| 3 | 1991 | Bio Controle | Thuricide | *Bacillus thuringiensis* var. kurstaki | *Ecdytolopha aurantiana, Diatraea saccharalis, Diaphania nitidalis, Diaphania hyalinata, Strymon basalides, Helicoverpa zea, Alabama argilacea, Ascia monuste orseis, Mocis latipes, Trichoplusia ni, Erinnyis ello, Colias lesbia pyrrhothea, Heliothis virescens, Anticarsia gemmatalis,*  *Brassolis sophorae, Brassolis astyra astyra, Opsiphanes invirae, Helicoverpa armigera, Dione juno juno, Eacles imperialis magnifica, Pseudoplusia includens, Rachiplusia nu, Spodoptera frugiperda, Thyrinteina arnobia, Manduca sexta paphus, Plutella xylostella* |
| 4 | 1995 | Bio Controle | Agree | *Bacillus thuringiensis aizawai* | *Grapholita molesta, Neoleucinodes elegante, Bonagota salubricola, Diaphania hyalinata, Spodoptera frugiperda, Chrysodeixis includens, Diaphania nitidalis, Ecdytolopha aurantian, Tuta absoluta, Plutella xylostella, Helicoverpa armigera, Cryptoblabes gnidiella* |
| 5 | 1998 | Mitsui | Able | *Bacillus thuringiensis* var. kurstaki | *Ecdytolopha aurantiana, Diaphania nitidalis, Ascia monuste orseis, Anticarsia gemmatalis, Brassolis sophorae, Helicoverpa armigera, Helicoverpa* sp*, Plutella xylostella, Tuta absoluta* |
| 6 | 1999 | Sumitomo | Xentari; Xtreem; | *Bacillus thuringiensis* | *Spodoptera frugiperda, Tuta absoluta, Ascia monuste orseis, Plutella xylostella* |
| 7 | 2001 | Sumitomo | Dipel WP | *Bacillus thuringiensis* var. kurstaki | *Helicoverpa* sp*, Strymon basalides, Colias lesbia pyrrothea, Opsiphanes invirae, Heliuothis virescens, Anticarsia gemmatalis, Ascia monuste orseis, Ascia monuste orseis, Mocis latipes, Pseudoplusia includens Spodoptera frugiperda, Brassolis astyra astyra, Brassolis sophorae Diaphania hyalinata, Diatraea saccharalis, Helicoverpa zea, Ecdytolopha aurantiana, Eacles imperialis magnifica, Plutella xylostella, Erinnyis ello, Trichoplusia ni, Manduca sexta paphus* |
| 8 | 2002 | Koppert | Boveril WP PL63 | *Beauveria bassiana* | *Bemisia tabaci* biotype B, *Tetranychus urticae, Hypothenemus hampei, Gonipterus scutellatus* |
| 9 | 2005 | Koppert | Metarril WP E9 | *Metarhizium anisopliae* | *Mahanarva fimbriolata* |
| 10 | 2007 | Sumitomo | *Dipel WG* | *Bacillus thuringiensis* var. kurstaki | *Alabama arqillacea, Grapholita molesta, Diaphania hyalinata, Plutella xylostella, Manduca sexta paphus, Erinnyis ello, Helicoverpa armigera, Chrysodeixis includens, Diatraea saccharalis, Argyrotaenia sphaleropa, Tuta absoluta, Anticarsia gemmatalis, Ecdytolopha aurantiana* |
| 11 | 2010 | Biotech | Biometha GR Plus | *Metarhizium anisopliae* | *Mahanarva fimbriolata, Zulia entreriana, Deois flavopicta* |
| 12 | 2010 | Toyobo | EcoMeta | *Metarhizium anisopliae* | *Mahanarva fimbriolata* |
| 13 | 2011 | Novozymes | Methamax EC | *Metarhizium anisopliae* | *Mahanarva fimbriolata* |
| 14 | 2011 | Novozymes | Bovemax EC | *Beauveria bassiana* | *Hypothenemus hampei, Diaphorina citri, Hedypathes betulinus* |
| 15 | 2012 | CEPLAC | Tricovab | *Trichoderma stromaticum* | *Moniliophthora perniciosa* |
| 16 | 2012 | Ballagro | Metiê; MetaNext; Methawin; Bionova Sanus Meta; | *Metarhizium anisopliae* | *Mahanarva fimbriolata, Zulia entreriana, Deois flavopicta* |
| 17 | 2012 | Ballagro | Ballvéria; Rizoveria Bio Inseticida; Mestrado; BoveNext; Bionova Sanus Bove; Beautop; | *Beauveria bassiana* | *Bemisia tabaci biotype B, Cosmopolites sordidus, Tetranychus urticae, Dalbulus maidis,Hypothenemus hampei, Gonipterus scutellatus, Coccus viridis Diaphorina citri* |
| 18 | 2013 | Companhia | Metarriz WP Biocontrol | *Metarhizium anisopliae* | *Mahanarva fimbriolata, Zulia entreriana* |
| 19 | 2013 | Bosquiroli e Santos | Baculovirus Soja WP | *Baculovirus Anticarsia* | *Anticarsia gemmatalis* |
| 20 | 2013 | Vittia | Meta-Turbo SC; Supremo; Meta-Guard; | *Metarhizium anisopliae* | *Mahanarva fimbriolata, Euschistus heros* |
| 21 | 2013 | Agrocete | Grap Baculovirus | *Baculovirus Anticarsia* | *Anticarsia gemmatalis* |
| 22 | 2014 | Fitoagro | Metarfito | *Metarhizium anisopliae* | *Mahanarva fimbriolata, Zulia entreriana, Deois flavopicta* |
| 23 | 2014 | Biofungi. | Bovebio | *Beauveria bassiana* | *Euschistus heros, Bemisia tabaci* biotype B*, Hypothenemus hampei* |
| 24 | 2015 | Biofungi | Metabiol | *Metarhizium anisopliae* | *Mahanarva fimbriolata, Zulia entreriana, Deois flavopicta* |
| 25 | 2015 | ASPLAN | Metarplan | *Metarhizium anisopliae* | *Mahanarva fimbriolata, Zulia entreriana, Deois flavopicta* |
| 26 | 2015 | Companhia Nitro Química | Metarriz GR Biocontrol | *Metarhizium anisopliae* | *Mahanarva fimbriolata, Zulia entreriana, Deois flavopicta* |
| 27 | 2015 | Mitsui | Gemstar-Max | *Helicoverpa zea* single nucleopolyhedrovirus | *Helicoverpa armigera* |
| 28 | 2015 | CCAB Agro | Hz-NPV CCAB | *Helicoverpa zea* single nucleopolyhedrovirus | *Helicoverpa armigera* |
| 29 | 2015 | Companhia Nitro Química | Bouveriz WP Biocontrol; Mirah; | *Beauveria bassiana* | *Bemisia tabaci biotype B, Cosmopolites sordidus, Tetranychus urticae, Dalbulus maidis, Sphenophorus levis* |
| 30 | 2015 | Agbitech | Armigen | *Helicoverpa zea* single nucleopolyhedrovirus | *Helicoverpa armigera* |
| 31 | 2015 | JCO | Metarhizium JCO | *Metarhizium anisopliae* | *Mahanarva fimbriolata, Zulia entreriana, Deois flavopicta* |
| 32 | 2015 | JCO | Beauveria JCO | *Beauveria bassiana* | *Bemisia tabaci biotype B, Cosmopolites sordidus, Tetranychus urticae, Dalbulus maidis, Sphenophorus levis* |
| 33 | 2015 | Genica | Paragran | *Metarhizium anisopliae* | *Mahanarva fimbriolata, Zulia entreriana, Deois flavopicta* |
| 34 | 2015 | Lallemand | Granada | *Beauveria bassiana* | *Bemisia tabaci biotype B, Cosmopolites sordidus, Tetranychus urticae, Dalbulus maidis, Sphenophorus levis* |
| 35 | 2015 | Companhia Nitro Química | Metarriz Plus WP Biocontrol; Titã; | *Metarhizium anisopliae* | *Mahanarva fimbriolata, Zulia entreriana, Deois flavopicta* |
| 36 | 2016 | Andermatt | Helicovex | *Baculovirus Condylorrhiza vestigialis* | *Helicoverpa armigera* |
| 37 | 2016 | Andermatt | Verpavex | *Baculovirus Condylorrhiza vestigialis* | *Helicoverpa armigera* |
| 38 | 2016 | Bioenergia | Biorhizium WP | *Metarhizium anisopliae* | *Mahanarva fimbriolata, Zulia entreriana, Deois flavopicta* |
| 39 | 2016 | Bioenergia | Biorhizium GR | *Metarhizium anisopliae* | *Mahanarva fimbriolata, Zulia entreriana, Deois flavopicta* |
| 40 | 2016 | Simbiose | MethaControl | *Metarhizium anisopliae* | *Mahanarva fimbriolata, Zulia entreriana, Deois flavopicta* |
| 41 | 2016 | Simbiose | BeauveControl | *Beauveria bassiana* | *Bemisia tabaci biotype B, Cosmopolites sordidus, Tetranychus urticae, Dalbulus maidis, Sphenophorus levis* |
| 42 | 2016 | Oligos Biotecnologia | Beauveria Oligos WP | *Beauveria bassiana* | *Bemisia tabaci biotype B, Cosmopolites sordidus, Tetranychus urticae, Dalbulus maidis, Sphenophorus levis* |
| 43 | 2016 | Simbiose | BTControl | *Bacillus thuringiensis* var. kurstaki | *Alabama arqillacea, Spodoptera frugiperda, Chrysodeixis includens, Anticarsia gemmatalis* |
| 44 | 2016 | Mitsui | Gemstar LC; Diplomata K; | *Helicoverpa zea* single nucleopolyhedrovirus | *Helicoverpa armigera* |
| 45 | 2016 | Oligos Biotecnologia | Metarhizium Oligos | *Metarhizium anisopliae* | *Mahanarva fimbriolata, Zulia entreriana, Deois flavopicta* |
| 46 | 2016 | Bioenergia | Bioveria WP | *Beauveria bassiana* | *Bemisia tabaci biotype B, Cosmopolites sordidus, Tetranychus urticae, Dalbulus maidis, Sphenophorus levis* |
| 47 | 2016 | Biopremium | Metapremium | *Metarhizium anisopliae* | *Mahanarva fimbriolata, Zulia entreriana, Deois flavopicta* |
| 48 | 2016 | Bionat | CartuchoVIT | *Spodoptera frugiperda* multiple nucleopolyhedrovirus | *Spodoptera frugiperda* |
| 49 | 2016 | PROBIO | Metarhizium Probio | *Metarhizium anisopliae* | *Mahanarva fimbriolata, Zulia entreriana, Deois flavopicta* |
| 50 | 2016 | Lallemand | Opala | *Metarhizium anisopliae* | *Mahanarva fimbriolata, Zulia entreriana, Deois flavopicta* |
| 51 | 2016 | TZ Biotec | Metarhyd | *Metarhizium anisopliae* | *Mahanarva fimbriolata, Zulia entreriana, Deois flavopicta* |
| 52 | 2016 | Vittia | Boveria-Turbo; BoveVale; Beauvebio; Brandt FlyShield; Accionfungi Boveria; SKHunter; | *Beauveria bassiana* | *Bemisia tabaci biotype B, Cosmopolites sordidus, Tetranychus urticae, Dalbulus maidis, Sphenophorus levis* |
| 53 | 2016 | Vittia | Meta-Turbo; Acciónfungi Meta; | *Metarhizium anisopliae* | *Mahanarva fimbriolata, Zulia entreriana, Deois flavopicta* |
| 54 | 2016 | Toyobo | Ecometa Power; Metasmart; Agoptimmon; Metatrix; Emeta Power; Vitales Gratto Meta; | *Metarhizium anisopliae* | *Mahanarva fimbriolata, Zulia entreriana, Deois flavopicta* |
| 55 | 2016 | Toyobo | Ecobass | *Beauveria bassiana* | *Bemisia tabaci biotype B, Cosmopolites sordidus, Tetranychus urticae, Dalbulus maidis, Sphenophorus levis* |
| 56 | 2016 | Andermatt | Helicovir | *Baculovirus Helicoverpa armigera* | *Helicoverpa armigera, Heliuothis virescens, Helicoverpa zea* |
| 57 | 2016 | Lallemand | Crystal | *Bacillus thuringiensis subsp. Thoworthy* | *Spodoptera frugiperda* |
| 58 | 2016 | Mitsui | Costar | *Bacillus thuringiensis* var. kurstaki | *Pseudaletia sequax, Grapholita molesta, Neoleucinodes elegantis, Plutella xylostella, Cryptoblabes gnidiella, Pseudaletia sequax, Ecdytolopha aurantiana, Helicoverpa armigera, Pseudoplusia includens, Diaphania nitidalis* |
| 59 | 2016 | TZ Biotec | Boveryd | *Beauveria bassiana* | *Bemisia tabaci biotype B, Cosmopolites sordidus, Tetranychus urticae, Dalbulus maidis, Sphenophorus levis* |
| 60 | 2016 | Agrivalle | Auin Cana WP | *Beauveria bassiana* | *Bemisia tabaci biotype B, Cosmopolites sordidus, Tetranychus urticae, Dalbulus maidis, Sphenophorus levis* |
| 61 | 2017 | Vectorcontrol | Bac-Control Max WP | *Bacillus thuringiensis* subsp. kurstaki, | *Ascia monuste orseis, Anticarsia gemmatalis, Eacles imperialis magnifica, Spodoptera frugiperda, Ecdytolopha aurantiana, Trichoplusia ni, Brassolis sophoroe, Thyrinteina arnobia, Heliuothis virescens, Manduca sexta paphus, Erinnvis ello, Helicoverpa armigera, Helicoverpa zea, Diaphania hyalinata, Diaphania nitidalis, Colias lesbia pyrrhothea, Alabama arqillacea, Plutella xylostella* |
| 62 | 2017 | Vectorcontrol | Tarik WP | *Bacillus thuringiensis, Berliner* | *Alabama arqillacea, Ascia monuste orseis, Ecdytolopha aurantiana, Erinnvis ello, Brassolis sophorae, Diaphania hyalinata, Diaphania nitidalis, Helicoverpa zea, Colias lesbia pyrrothea,, Anticarsia gemmatalis, Helicoverpa armigera, Heliuothis virescens, Mocis latipes, Spodoptera frugiperda, Thyrinteina arnobia, Trichoplusia ni, Plutella xylostella, Manduca sexta paphus* |
| 63 | 2017 | Sumitomo | Dipel ES-NT; Biobit; Quark; Bactospeine; | *Bacillus thuringiensis var. kurstak* | *Chrysodeixis includens, Pseudaletia sequax, Helicoverpa armigera, Trichoplusia ni, Tuta absoluta, Alabama arqillacea, Ascia monuste orseis, Diatraea saccharalis, Opsiphanes invirae, Thyrinteina arnobia, Heliuothis virescens, Condylorrhiza vestigialis, Brassolis sophorae, Anticarsia gemmatalis, Ecdytolopha aurantiana* |
| 64 | 2017 | JCO | Metarhizium JCO WP | *Metarhizium anisopliae* | *Mahanarva fimbriolata, Zulia entreriana, Deois flavopicta* |
| 65 | 2017 | Agrivalle | Gr-Inn; Bio Brado; | *Metarhizium anisopliae* | *Mahanarva fimbriolata, Zulia entreriana, Deois flavopicta* |
| 66 | 2017 | Koppert | KBR-H19S3 | *Baculovirus helicorpa armigera* | *Helicoverpa armigera* |
| 67 | 2017 | Mitsui | Javelin WG | *Bacillus thuringiensis* var. kurstaki | *Helicoverpa armigera, Pseudoplusia includens* |
| 68 | 2017 | Koppert | Corvair | *Beauveria bassiana* | *Bemisia tabaci biotype B, Cosmopolites sordidus, Tetranychus urticae, Dalbulus maidis, Sphenophorus levis* |
| 69 | 2017 | Koppert | Challenger | *Isaria fumosorosea* | *Diaphorina citri, Helicoverpa armigera* |
| 70 | 2017 | Koppert | Octane | *Isaria fumosorosea* | *Diaphorina citri, Helicoverpa armigera, Dalbulus maidis* |
| 71 | 2017 | Ballagro | Helymax EC; Bionic; BT Top; | *Bacillus thuringiensis* | *Ecdytolopha aurantiana, Helicoverpa armigera, Thyrinteina arnobia, Helicoverpa zea, Plutella xylostella* |
| 72 | 2017 | Koppert | Metatec | *Metarhizium anisopliae* | *Mahanarva fimbriolata, Zulia entreriana, Deois flavopicta* |
| 73 | 2017 | Koppert | Detroit | *Metarhizium anisopliae* | *Mahanarva fimbriolata, Zulia entreriana, Deois flavopicta* |
| 74 | 2017 | Koppert | Atrevido | *Beauveria bassiana* | *Bemisia tabaci biotype B, Cosmopolites sordidus, Tetranychus urticae, Dalbulus maidis, Sphenophorus levis* |
| 75 | 2017 | Koppert | Boveril Cana | *Beauveria bassiana* | *Bemisia tabaci biotype B, Cosmopolites sordidus, Tetranychus urticae, Dalbulus maidis, Sphenophorus levis* |
| 76 | 2017 | Nutrien | Arcar | *Metarhizium anisopliae* | *Mahanarva fimbriolata, Zulia entreriana, Deois flavopicta* |
| 77 | 2017 | Mitsui | Thuricide SC; Costar SC; | *Bacillus thuringiensis* var. kurstaki | *Helicoverpa armigera, Anticarsia gemmatalis, Chysodeixis includens, Thyrinteina arnobia, Trichoplusia ni, Tuta absoluta, Pseudaletia sequax, Alabama argilacea, Mocis latipes, Diaphania hyalinata, Condylorrhiza vestigialis* |
| 78 | 2017 | Vectorcontrol | Tarik EC; BTvale; Cepakill; QuestBT; BS Beta; Agrofreedom BT; | *Bacillus thuringiensis* var. kurstaki | *Ecdytolopha aurantiana, Thyrinteina arnobia, Plutella xylostella, Spodoptera frugiperda, Helicoverpa zea, Erinnyis ello, Helicoverpa armigera* |
| 79 | 2017 | Simbiose | Vircontrol S.F | *Baculovirus Spodoptera frugiperda* | Spodoptera frugiperda |
| 80 | 2017 | Vital Brasil | BioBVB | *Beauveria bassiana* | *Bemisia tabaci biotype B, Cosmopolites sordidus, Tetranychus urticae, Dalbulus maidis, Sphenophorus levis* |
| 81 | 2018 | Simbiose | Biagro Attak | *Beauveria bassiana* | *Bemisia tabaci biotype B, Cosmopolites sordidus, Tetranychus urticae, Dalbulus maidis, Sphenophorus levis* |
| 82 | 2018 | Vectorcontrol | Winner Max EC | *Bacillus thuringiensis* var. kurstaki | *Ecdytolopha aurantiana, Helicoverpa armigera, Thyrinteina arnobia, Plutella xylostella, Helicoverpa zea* |
| 83 | 2018 | União Química | Ponto Final | *Bacillus thuringiensis* var. kurstaki | *Spodoptera frugiperda, Anticarsia gemmatalis, Pseudoplusia includens* |
| 84 | 2018 | Agbitech | Chrysogen | *Baculovirus Chrysodeixis includens* | *Chrysodeixis includens* |
| 85 | 2018 | Agbitech | Chrysogen CCAB | *Baculovirus Chrysodeixis includens* | *Chrysodeixis includens* |
| 86 | 2018 | Agbitech | Cartugen | *Spodoptera frugiperda* multiple nucleopolyhedrovirus | *Spodoptera frugiperda* |
| 87 | 2018 | Agbitech | Sfmnpv ABT 01 | *Baculovirus - Spodoptera frugiperda* multiple nucleopolyhedrovirus | *Spodoptera frugiperda* |
| 88 | 2018 | Simbiose | Biagro Cristalles | *Bacillus thuringiensis* var. kurstaki | *Anticarsia gemmatalis, Chrysodeixis includens, Helicoverpa armigera* |
| 89 | 2018 | Agbitech | Surtivo Soja | *Baculovirus Chrysodeixis includens + Baculovirus Helicoverpa armigera* | *Helicoverpa armigera, Pseudoplusia includens* |
| 90 | 2018 | Agrobiológica | Aptur-PF | *Paecilomyces fumosoroseus* | *Dalbulus maidis, Bemisia tabaci* |
| 91 | 2018 | Agbitech | Disseminate | *Autographa californica* multiple nucleopolyhedrovirus | *Chrysodeixis includens, Helicoverpa armigera, Spodoptera eridania* |
| 92 | 2018 | Agrivalle | Auin CE; Bio Tribal; Goveria Goplan; | *Beauveria bassiana* | *Diabrotica speciosa* |
| 93 | 2018 | Ballagro | Hakun | *Metarhizium anisopliae* | *Mahanarva fimbriolata, Zulia entreriana, Deois flavopicta* |
| 94 | 2018 | TZ Biotec | Boveryd FR 25 | *Beauveria bassiana* | *Bemisia tabaci* |
| 95 | 2018 | Agrobiológica | Dobbel | *Beauveria bassiana + Metarhizium anisopliae* | *Deois flavopicta, Euschistus heros* |
| 96 | 2018 | Ballagro | Dux; Biotrinsic Beauveria; | *Beauveria bassiana* | *Bemisia tabaci biotype B, Cosmopolites sordidus, Tetranychus urticae, Dalbulus maidis, Sphenophorus levis* |
| 97 | 2018 | Vectorcontrol | Bac Control Max EC | *Bacillus thuringiensis* subsp. kurstaki, | *Ecdytolopha aurantiana, Helicoverpa armigera, Thyrinteina arnobia, Helicoverpa zea, Plutella xylostella* |
| 98 | 2018 | Excellence | Excellence Rugger | *Metarhizium anisopliae* | *Mahanarva fimbriolata, Zulia entreriana, Deois flavopicta* |
| 99 | 2018 | Excellence | Excellence Mig-66 | *Beauveria bassiana* | *Bemisia tabaci biotype B, Cosmopolites sordidus, Tetranychus urticae, Dalbulus maidis, Sphenophorus levis* |
| 100 | 2019 | Vittia | BT-Turbo Max; BT-Guard; BT-Turbo; Power BAC BT; | *Bacillus thuringiensis* var. kurstaki | *Chrysodeixis includens, Thyrinteina arnobia, Ecdytolopha aurantiana* |
| 101 | 2019 | TZ Biotec | Metarhyd FR 25 | *Metarhizium anisopliae* | *Mahanarva fimbriolata* |
| 102 | 2019 | Simbiose | Beauvecontrol Extreme | *Beauveria bassiana* | *Bemisia tabaci biotype B, Hypothenemus hampei* |
| 103 | 2019 | Oligos | Metarhizium Oligos WP | *Metarhizium anisopliae* | *Mahanarva fimbriolata, Zulia entreriana, Deois flavopicta* |
| 104 | 2019 | Vectorcontrol | Stregga EC | *Bacillus thuringiensis* var. kurstaki | *Ecdytolopha aurantiana, Helicoverpa armigera, Thyrinteina arnobia, Plutella xylostella, Helicoverpa zea* |
| 105 | 2019 | Dillon | Beauvel | *Beauveria bassiana* | *Diabrotica speciosa* |
| 106 | 2019 | Agropaulo | Nat Fungi | *Metarhizium anisopliae* | *Mahanarva fimbriolata, Zulia entreriana, Deois flavopicta* |
| 107 | 2019 | Serquibio | Biobev; Cropbio BioVeria; | *Beauveria bassiana* | *Diabrotica speciosa* |
| 108 | 2019 | TOPBIO | Bravo | *Beauveria bassiana* | *Bemisia tabaci biotype B, Cosmopolites sordidus, Tetranychus urticae, Dalbulus maidis, Sphenophorus levis* |
| 109 | 2019 | Bionat | Metarhizonat | *Metarhizium anisopliae* | *Mahanarva fimbriolata, Zulia entreriana, Deois flavopicta* |
| 110 | 2019 | Ballagro | Bometil; Beaumetha; | *Beauveria bassiana + Metarhizium anisopliae* | *Deois flavopicta, Euschistus heros, Bemisia tabaci biotype B, Tetranychus urticae, Sphenophorus levis, Hypothenemus hampei, Frankliniella occidentalis* |
| 111 | 2019 | Agrobiológica | Álaabo; Isatrix; Isashock; | *Paecilomyces fumosoroseus* | *Dalbulus maidis, Bemisia tabaci* |
| 112 | 2019 | Mitsui | Mycotrol ES | *Beauveria bassiana* | *Bemisia tabaci* biotype B *, Frankliniella occidentalis, Hypothenemus hampei* |
| 113 | 2019 | Bionat | Bovenat | *Beauveria bassiana* | *Bemisia tabaci biotype B, Cosmopolites sordidus, Tetranychus urticae, Dalbulus maidis, Sphenophorus levis* |
| 114 | 2019 | Ballagro | BI2003/16 | *Beauveria bassiana + Metarhizium anisopliae* | *Deois flavopicta, Euschistus heros* |
| 115 | 2019 | Agrivalle | Sulis CE | *Beauveria bassiana* | *Diabrotica speciosa* |
| 116 | 2019 | Basf | Vestix | *Beauveria bassiana* | *Bemisia tabaci* biotype B |
| 117 | 2019 | TOPBIO | Optimun | *Metarhizium anisopliae* | *Mahanarva fimbriolata, Zulia entreriana, Deois flavopicta* |
| 118 | 2020 | Genica | Aradya | *Metarhizium anisopliae* | *Mahanarva fimbriolata, Zulia entreriana, Deois flavopicta* |
| 119 | 2020 | COMDEAGRO | Bacmix BTKSC | *Bacillus thutingiensis* var. kurstaki | *Alabama arqillacea, Chrysodeixis includens, Euschistus heros, Anticarsia gemmatalis, Pseudoplusia includens* |
| 120 | 2020 | Innova | Bassi Control | *Beauveria bassiana* | *Bemisia tabaci biotype B, Cosmopolites sordidus, Tetranychus urticae, Dalbulus maidis, Sphenophorus levis* |
| 121 | 2020 | Serquibio | BtFert; Cropbio BioThur; | *Bacillus thuringiensis* var. kurstaki | *Alabama arqillacea, Spodoptera frugiperda, Chrysodeixis includens, Anticarsia gemmatalis* |
| 122 | 2020 | Innova | Green Muscardine | *Metarhizium anisopliae* | *Mahanarva fimbriolata, Zulia entreriana, Deois flavopicta* |
| 123 | 2020 | Biomip | Biometa | *Metarhizium anisopliae* | *Mahanarva fimbriolata, Zulia entreriana, Deois flavopicta* |
| 124 | 2020 | Prophyto | BMS MAX; movB; | *Beauveria bassiana + Metarhizium anisopliae* | *Deois flavopicta, Euschistus heros* |
| 125 | 2020 | Nooa | Bovettus Org; Vitales Gratto Bovers; | *Beauveria bassiana* | *Bemisia tabaci biotype B, Cosmopolites sordidus, Tetranychus urticae, Dalbulus maidis, Sphenophorus levis* |
| 126 | 2020 | Genica | Latria | *Beauveria bassiana* | *Bemisia tabaci biotype B, Cosmopolites sordidus, Tetranychus urticae, Dalbulus maidis, Sphenophorus levis* |
| 127 | 2020 | Simbiose | VirControl C.I | *Chrysodeixis Includens nucleopolyhedrovirus* | *Chrysodeixis includens, Rachiplusia nu* |
| 128 | 2020 | Dillon | Mesopel Mix; Easy Spir; | *Beauveria bassiana + Metarhizium anisopliae* | *Deois flavopicta, Euschistus heros* |
| 129 | 2020 | Biomip | BioBassi | *Beauveria bassiana* | *Bemisia tabaci* biotype B , *Cosmopolites sordidus, Tetranychus urticae, Dalbulus maidis* |
| 130 | 2020 | Nooa | Mettus Org; Vitales Gratto Metarz; | *Metarhizium anisopliae* | *Mahanarva fimbriolata, Zulia entreriana, Deois flavopicta* |
| 131 | 2020 | Bioma | Vir Protection | *Spodoptera frugiperda* multiple nucleopolyhedrovirus | *Spodoptera frugiperda* |
| 132 | 2020 | Ballagro | Acera | *Bacillus thuringiensis* | *Spodoptera frugiperda, Chrysodeixis includens* |
| 133 | 2020 | Ballagro | BI73.002/17 | *Bacillus thuringiensis* | *Spodoptera frugiperda, Chrysodeixis includens* |
| 134 | 2020 | Simbiose | IsaControl | *Isaria fumosorosea* | *Bemisia tabaci* biotype B, *Diaphorina citri* |
| 135 | 2020 | Simbiose | Methacontrol Evolution | *Metarhizium anisopliae* | *Scaptocoris castânea* |
| 136 | 2020 | Agbitech | Biodeixis | *Chrysodeixis includens nucleopolyhedrovirus* | *Chrysodeixis includens* |
| 137 | 2020 | Koppert | Boveril Evo | *Beauveria bassiana* | *Euschistus heros*, *Bemisia tabaci* biotype B, *Hypothenemus hampei* |
| 138 | 2020 | Sumitomo | Sympatico | *Bacillus thuringiensis* subsp. Kurstaki + *Bacillus thuringiensis* subsp. Aizawai | *Spodoptera frugiperda, Pseudoplusia includens, Spodoptera eridania* |
| 139 | 2020 | Koppert | Buick | *Spodoptera frugiperda* multiple nucleopolyhedrovirus | *Spodoptera frugiperda* |
| 140 | 2020 | JCO | Biomatch JCO | *Beauveria bassiana + Metarhizium anisopliae* | *Deois flavopicta, Euschistus heros* |
| 141 | 2020 | Agrobiológica | Assertive; Duotrix; | *Beauveria bassiana + Metarhizium anisopliae* | *Deois flavopicta, Euschistus heros* |
| 142 | 2020 | Koppert | Boveril Plus | *Beauveria bassiana* | *Euschistus heros*, *Bemisia tabaci* biotype B, *Hypothenemus hampei* |
| 143 | 2020 | Promip | Bovemip | *Beauveria bassiana* | *Bemisia tabaci biotype B, Cosmopolites sordidus, Tetranychus urticae, Dalbulus maidis, Sphenophorus levis* |
| 144 | 2020 | Promip | Metamip | *Metarhizium anisopliae* | *Mahanarva fimbriolata, Zulia entreriana, Deois flavopicta* |
| 145 | 2020 | Vittia S.A | Bovéria-Guard | *Beauveria bassiana* | *Bemisia tabaci biotype B, Cosmopolites sordidus, Tetranychus urticae, Dalbulus maidis, Sphenophorus levis* |
| 146 | 2020 | Massen | Sumério; Vitanica Bug Protect; Biogreat; Tackler; Goveria; | *Beauveria bassiana* | *Diabrotica speciosa* |
| 147 | 2020 | Vital Brasil | Bioisa | *Isaria fumosorosea* | *Bemisia tabaci* biotype B |
| 148 | 2020 | Oligos | Beauveria SR | *Beauveria bassiana* | *Bemisia tabaci biotype B, Cosmopolites sordidus, Tetranychus urticae, Dalbulus maidis, Sphenophorus levis* |
| 149 | 2020 | Lallemand | Opala WP | *Metarhizium anisopliae* | *Mahanarva fimbriolata, Zulia entreriana, Deois flavopicta* |
| 150 | 2020 | Koppert | Bove K | *Beauveria bassiana* | *Euschistus heros*, *Bemisia tabaci* biotype B, *Hypothenemus hampei* |
| 151 | 2020 | Vital Brasil | Bioscap | *Beauveria bassiana* | *Euschistus heros*, *Bemisia tabaci* biotype B |
| 152 | 2020 | Vital Brasil | Trapper | *Beauveria bassiana* | *Bemisia tabaci* biotype B, *Cosmopolites sordidus, Tetranychus urticae, Dalbulus maidis* |
| 153 | 2020 | Simbiose | FlyControl | *Beauveria bassiana* | *Euschistus heros*, *Bemisia tabaci* biotype B |
| 154 | 2020 | Vittia S.A. | Bovéria-Turbo SC; BassMax; Bio-Bass; | *Beauveria bassiana* | *Bemisia tabaci* biotype B*, Tetranychus urticae, Dalbulus maidis, Hypothenemus hampei* |
| 155 | 2020 | Bioma | Beauve Protection; Rizovéria; | *Beauveria bassiana* | *Bemisia tabaci* biotype B*, Tetranychus urticae, Dalbulus maidis, Sphenophorus levis* |
| 156 | 2021 | Promip | Baculomip-SF; Spinix; BioCash; Baculoshock; | *Spodoptera frugiperda* multiple nucleopolyhedrovirus | *Spodoptera frugiperda* |
| 157 | 2021 | Nooa | Bettus Org; Vitales Gratto Turi; BT-Ouro; | *Bacillus thuringiensis var.* kurstaki | *Alabama arqillacea, Spodoptera frugiperda, Chrysodeixis includens, Anticarsia gemmatalis* |
| 158 | 2021 | Oligos | Metarhizium SR | *Metarhizium anisopliae* | *Mahanarva fimbriolata, Zulia entreriana, Deois flavopicta* |
| 159 | 2021 | JCO | Btkill JCO | *Bacillus thuringiensis* var. kurstaki | *Alabama arqillacea, Spodoptera frugiperda, Chrysodeixis includens, Anticarsia gemmatalis* |
| 160 | 2021 | Bioma | Metha Protection | *Metarhizium anisopliae* | *Mahanarva fimbriolata, Zulia entreriana, Deois flavopicta* |
| 161 | 2021 | Bionat | Baculonat SF | *Spodoptera frugiperda* multiple nucleopolyhedrovirus | *Spodoptera frugiperda* |
| 162 | 2021 | Endeavour | Biossela | *Bacillus thuringiensis* var. aizawai | *Spodoptera frugiperda, Chrysodeixis includens, Diatraea saccharalis* |
| 163 | 2021 | Andermatt | Spodovir | *Spodoptera littoralis nucleopolyhedrovirus* | *Spodoptera frugiperda* |
| 164 | 2021 | Biota | Boven; Questbeauve; BS Guard; Bouver; Beauguard; Fortify; | *Beauveria bassiana* | *Bemisia tabaci biotype B, Cosmopolites sordidus, Tetranychus urticae, Dalbulus maidis, Sphenophorus levis* |
| 165 | 2021 | Vital Brasil | BioBac T | *Bacillus thuringiensis* var. kurstaki | *Alabama arqillacea, Spodoptera frugiperda, Chrysodeixis includens, Anticarsia gemmatalis* |
| 166 | 2021 | Simbiose | FlyControl WP | *Beauveria bassiana* | *Euschistus heros*, *Bemisia tabaci* biotype B, *Hypothenemus hampei* |
| 167 | 2021 | Mitsui & Co | Botanigard WP | *Beauveria bassiana* | *Bemisia tabaci biotype B, Cosmopolites sordidus, Tetranychus urticae, Hypothenemus hampei, Diaphorina citri, Thrips tabaci* |
| 168 | 2021 | Biomip | Tbio | *Beauveria bassiana + Metarhizium anisopliae* | *Dalbulus maidis, Euschistus heros* |
| 169 | 2021 | Andermatt | Loopovir | *Chrysodeixis includens nucleopolyhedrovirus* | *Chrysodeixis includens* |
| 170 | 2021 | COMDEAGRO | Bemitrix WG | *Beauveria bassiana* | *Bemisia tabaci biotype B, Cosmopolites sordidus, Tetranychus urticae, Dalbulus maidis, Sphenophorus levis* |
| 171 | 2021 | Agropaulo | Nat Beauveria | *Beauveria bassiana* | *Bemisia tabaci biotype B, Cosmopolites sordidus, Tetranychus urticae, Dalbulus maidis, Sphenophorus levis* |
| 172 | 2021 | COMDEAGRO | Metamix WG | *Metarhizium anisopliae* | *Mahanarva fimbriolata, Zulia entreriana, Deois flavopicta* |
| 173 | 2021 | Vital Brasil | Bioscap Liq | *Beauveria bassiana + Metarhizium anisopliae* | *Deois flavopicta, Euschistus heros* |
| 174 | 2021 | Vital Brasil | Ecobals | *Beauveria bassiana* | *Bemisia tabaci biotype B, Cosmopolites sordidus, Tetranychus urticae, Dalbulus maidis, Sphenophorus levis* |
| 175 | 2021 | Vital Brasil | Ecobaci T | *Bacillus thuringiensis* var. kurstaki | *Alabama arqillacea, Spodoptera frugiperda, Chrysodeixis includens, Anticarsia gemmatalis* |
| 176 | 2021 | Vital Brasil | Ecotetran | *Beauveria bassiana + Metarhizium anisopliae* | *Deois flavopicta, Euschistus heros* |
| 177 | 2021 | Bioma | Looper Protection | *Chrysodeixis includens nucleopolyhedrovirus* | *Chrysodeixis includens* |
| 178 | 2021 | Simbiose | Elagesk C.I. | *Chrysodeixis includens nucleopolyhedrovirus* | *Chrysodeixis includens, Rachiplusia nu* |
| 179 | 2021 | Vital Brasil | Ecocordy | *Isaria fumosorosea* | *Bemisia tabaci* biotype B |
| 180 | 2021 | Total Biotecnologia | Bioatena | *Metarhizium anisopliae* | *Mahanarva fimbriolata, Zulia entreriana, Deois flavopicta* |
| 181 | 2021 | Simbiose | VirControl H.A. | *Baculovirus Helicoverpa armigera* | *Helicoverpa armigera* |
| 182 | 2021 | CL Empreendimentos Biológicos | Destroyer; Revers; Virusguard; Voliturcho; Gratto Frugi; | *Spodoptera frugiperda* multiple nucleopolyhedrovirus | *Spodoptera frugiperda* |
| 183 | 2021 | Bioma | BT Protection | *Bacillus thuringiensis* var. kurstaki | *Alabama arqillacea, Spodoptera frugiperda, Chrysodeixis includens, Anticarsia gemmatalis* |
| 184 | 2021 | Total Biotecnologia | Bioolimpo; BiologiC Bravia; | *Beauveria bassiana + Metarhizium anisopliae* | *Deois flavopicta, Euschistus heros* |
| 185 | 2021 | Total Biotecnologia | BTP 078-20 | *Beauveria bassiana + Metarhizium anisopliae* | *Deois flavopicta, Euschistus heros* |
| 186 | 2021 | Genica | GNC 009-2 | *Metarhizium anisopliae* | *Mahanarva fimbriolata, Zulia entreriana, Deois flavopicta* |
| 187 | 2021 | Toyobo | Ecobass Ultra; Beauvesmart; Agnexmmon; Bovetrix; Vitales Gratto Bover; Evomult; | *Beauveria bassiana* | *Bemisia tabaci biotype B, Cosmopolites sordidus, Tetranychus urticae, Dalbulus maidis, Sphenophorus levis* |
| 188 | 2021 | Total Biotecnologia | Biosparta | *Beauveria bassiana* | *Bemisia tabaci biotype B, Cosmopolites sordidus, Tetranychus urticae, Dalbulus maidis, Sphenophorus levis* |
| 189 | 2021 | Toyobo | Mahazai | *Metarhizium anisopliae* | *Mahanarva fimbriolata, Zulia entreriana, Deois flavopicta* |
| 190 | 2021 | Toyobo | Nomite | *Beauveria bassiana* | *Bemisia tabaci biotype B, Cosmopolites sordidus, Tetranychus urticae, Dalbulus maidis, Sphenophorus levis* |
| 191 | 2021 | Total Biotecnologia | BTP 076-20 | *Metarhizium anisopliae* | *Mahanarva fimbriolata, Zulia entreriana, Deois flavopicta* |
| 192 | 2021 | Total Biotecnologia | BTP 077-20 | *Beauveria bassiana* | *Bemisia tabaci biotype B, Cosmopolites sordidus, Tetranychus urticae, Dalbulus maidis, Sphenophorus levis* |
| 193 | 2021 | Vital Brasil | Isa 3 | *Isaria fumosorosea* | *Bemisia tabaci* biotype B |
| 194 | 2021 | Simbiose | Drill Protection | *Beauveria bassiana* | *Bemisia tabaci* biotype B*, Hypothenemus hampei* |
| 195 | 2022 | Genica | Papillon | *Beauveria bassiana* | *Bemisia tabaci biotype B, Cosmopolites sordidus, Tetranychus urticae, Dalbulus maidis, Sphenophorus levis* |
| 196 | 2022 | Ecosolução | Squadra* | *Beauveria bassiana* | *Bemisia tabaci* biotype B*, Hypothenemus hampei* |
| 197 | 2022 | Bom Futuro | Bacillus Thuringiensis Bom Futuro | *Bacillus thuringiensis* var. kurstaki | *Alabama arqillacea, Spodoptera frugiperda, Chrysodeixis includens, Anticarsia gemmatalis* |
| 198 | 2022 | Oligos | Beauve 100; Titanium Bove; | *Beauveria bassiana* | *Bemisia tabaci biotype B, Cosmopolites sordidus, Tetranychus urticae, Dalbulus maidis, Sphenophorus levis* |
| 199 | 2022 | Oligos | Hizium 100; Titanium Meta; | *Metarhizium anisopliae* | *Mahanarva fimbriolata, Zulia entreriana, Deois flavopicta* |
| 200 | 2022 | Biossíntese | Bovax | *Beauveria bassiana* | *Bemisia tabaci biotype B, Cosmopolites sordidus, Tetranychus urticae, Dalbulus maidis, Sphenophorus levis* |
| 201 | 2022 | Biossíntese. | Methabio | *Metarhizium anisopliae* | *Mahanarva fimbriolata, Zulia entreriana, Deois flavopicta* |
| 202 | 2022 | Solubio | Tec Finish; Solo Tec; | *Metarhizium anisopliae* | *Mahanarva fimbriolata, Zulia entreriana, Deois flavopicta* |
| 203 | 2022 | Solubio | Tec White; Air Tec; Solo Tec; | *Beauveria bassiana* | *Bemisia tabaci biotype B, Cosmopolites sordidus, Tetranychus urticae, Dalbulus maidis, Sphenophorus levis* |
| 204 | 2022 | Elo | Combat MBC | *Metarhizium anisopliae* | *Mahanarva fimbriolata, Zulia entreriana, Deois flavopicta* |
| 205 | 2022 | Simbiose | Tezpetix Beauve | *Beauveria bassiana* | *Hypopthenemus hampei, Bemisia tabaci* biotype B*, Dalbulus maidis, Euchistus heros, Dichlops melacanthus* |
| 206 | 2022 | Simbiose | Elagesk S.f | *Spodoptera frugiperda* multiple nucleopolyhedrovirus | *Spodoptera frugiperda* |
| 207 | 2022 | Solubio | Tec Catp Pro | *Bacillus thuringiensis* var. kurstaki | *Alabama arqillacea, Spodoptera frugiperda, Chrysodeixis includens, Anticarsia gemmatalis* |
| 208 | 2022 | Genica | GNC 009-3 | *Metarhizium anisopliae* | *Mahanarva fimbriolata, Zulia entreriana, Deois flavopicta* |
| 209 | 2022 | Elo | Fulminante AKb | *Beauveria bassiana* | *Bemisia tabaci* biotype B*, Cosmopolites sordidus, Tetranychus urticae, Dalbulus maidis, Sphenophorus levis* |
| 210 | 2022 | Bioma | Vector Protection | *Beauveria bassiana* | *Bemisia tabaci* biotype B*, Dalbulus maidis, Euchistus heros* |
| 211 | 2022 | Bioma | Biagro Pasto | *Metarhizium anisopliae* | *Mahanarva fimbriolata, Zulia entreriana, Deois flavopicta* |
| 212 | 2022 | Bionat | Bio Phygga | *Metarhizium anisopliae* | *Mahanarva fimbriolata, Zulia entreriana, Deois flavopicta* |
| 213 | 2022 | Total Biotecnologia | Biokato; Bioddam; | *Pseudomonas fluorescens + Pseudomonas chlororaphis* | *Caliothrips brasiliensis, Frankliniella schultzei Rhopalosiphum graminum, Aphis gossypii, Dichlops melacanthus,Sphenophorus levis, Diaphorina citri, Tetranychus urticae, Dalbulus maidis, Euchistus heros, Bemisia tabaci, Leucoptera coffeella* |
| 214 | 2022 | Genica | GNC 010-3 | *Beauveria bassiana* | *Bemisia tabaci* biotype B*, Cosmopolites sordidus, Tetranychus urticae, Dalbulus maidis, Sphenophorus levis* |
| 215 | 2022 | Vital Brasil | BioMTZ | *Metarhizium anisopliae* | *Mahanarva fimbriolata, Zulia entreriana, Deois flavopicta* |
| 216 | 2022 | Vital Brasil | EcoClavy | *Metarhizium anisopliae* | *Mahanarva fimbriolata, Zulia entreriana, Deois flavopicta* |
| 217 | 2022 | Macrobio | Raubtier | *Beauveria bassiana + Metarhizium anisopliae* | *Deois flavopicta, Euschistus heros* |
| 218 | 2022 | Total Biotecnologia | Aevo | *Pseudomonas fluorescens + Pseudomonas chlororaphis* | *Caliothrips brasiliensis, Frankliniella schultzei Rhopalosiphum graminum, Aphis gossypii, Dichlops melacanthus,Sphenophorus levis, Diaphorina citri, Tetranychus urticae, Dalbulus maidis, Euchistus heros, Bemisia tabaci, Leucoptera coffeella* |
| 219 | 2022 | Simbiose | Biagro Impacto | *Isaria fumosorosea* | *Bemisia tabaci* biotype B, *Diaphorina citri* |
| 220 | 2022 | Total Biotecnologia | Takotrop | *Pseudomonas fluorescens + Pseudomonas chlororaphis* | *Caliothrips brasiliensis, Frankliniella schultzei Rhopalosiphum graminum, Aphis gossypii, Dichlops melacanthus, Sphenophorus levis, Diaphorina citri, Tetranychus urticae, Dalbulus maidis, Euchistus heros, Bemisia tabaci, Leucoptera coffeella* |
| 221 | 2022 | Bioma | Biagro Cana | *Metarhizium anisopliae* | *Mahanarva fimbriolata, Zulia entreriana, Deois flavopicta* |
| 222 | 2022 | Bioma | Leprotect S.F. | *Spodoptera frugiperda* multiple nucleopolyhedrovirus | *Spodoptera frugiperda* |
| 223 | 2022 | Bom Futuro | Beauveria Bom Futuro | *Beauveria bassiana* | *Bemisia tabaci* biotype B*, Cosmopolites sordidus, Tetranychus urticae, Dalbulus maidis, Sphenophorus levis* |
| 224 | 2022 | VSF | Concriz | *Beauveria bassiana* | *Bemisia tabaci* biotype B*, Cosmopolites sordidus, Tetranychus urticae, Dalbulus maidis, Sphenophorus levis* |
| 225 | 2022 | Bionat | Bio Centules | *Beauveria bassiana* | *Bemisia tabaci* biotype B*, Cosmopolites sordidus, Tetranychus urticae, Dalbulus maidis, Sphenophorus levis* |
| 226 | 2022 | Lallemand | Lalguard C99 WP | Cordyceps javanica | *Bemisia tabaci* biotype B |
| 227 | 2022 | Andermatt | Tutavir | *hthorimaea operculella granulovirus* | *Tuta* *absoluta, Phthorimaea operculella* |
| 228 | 2022 | Agrobiológica | Epizotil | *Metarhizium rileyi* | *Spodoptera frugiperda* |
| 229 | 2022 | Hubio Biopar | Modera | *Metarhizium anisopliae* | *Mahanarva fimbriolata, Zulia entreriana, Deois flavopicta* |
| 230 | 2022 | COMDEAGRO | Virumix WP | *Baculovirus Spodoptera frugiperda* | *Spodoptera frugiperda* |
| 231 | 2022 | Bionat | Lepthure; Bio Taurus; Tuiuiu; Devorador Complex; | *Bacillus thuringiensis* | *Anticarsia gemmatalis, Spodoptera frugiperda, Chrysodeixis includens* |
| 232 | 2022 | Agrobiológica | Spodomax | *Metarhizium rileyi* | *Spodoptera frugiperda* |
| 233 | 2022 | Viva | BovCombat | *Beauveria bassiana* | *Bemisia tabaci* biotype B*, Cosmopolites sordidus, Tetranychus urticae, Dalbulus maidis, Sphenophorus levis* |
| 234 | 2022 | Viva | Metacombat | *Metarhizium anisopliae* | *Mahanarva fimbriolata, Zulia entreriana, Deois flavopicta* |
| 235 | 2022 | Prophyto | Turin-Cry S | *Bacillus thuringiensis* var. kurstaki | *Alabama arqillacea, Spodoptera frugiperda, Chrysodeixis includens, Anticarsia gemmatalis* |
| 236 | 2022 | Solatus | Batuk; Kubera; | *Beauveria bassiana* | *Bemisia tabaci* biotype B*, Cosmopolites sordidus, Tetranychus urticae, Dalbulus maidis, Sphenophorus levis* |
| 237 | 2022 | Solatus | Umet | *Metarhizium anisopliae* | *Mahanarva fimbriolata, Zulia entreriana, Deois flavopicta* |
| 238 | 2022 | Dillon | Btel | *Bacillus thuringiensis* | *Plutella xystola, Helicoverpa armigera* |
| 239 | 2022 | Bom Futuro | Metarhizium Anisopliae Bom Futuro | *Metarhizium anisopliae* | *Mahanarva fimbriolata, Zulia entreriana, Deois flavopicta* |
| 240 | 2022 | Bom Futuro | Bove-Meta Bom Futuro | *Beauveria bassiana + Metarhizium anisopliae* | *Dalbulus maidis, Euschistus heros* |
| 241 | 2022 | Valeouro | BeauveOuro | *Beauveria bassiana* | *Bemisia tabaci* biotype B*, Cosmopolites sordidus, Tetranychus urticae, Dalbulus maidis, Sphenophorus levis* |
| 242 | 2022 | Valeouro | MethaOuro | *Metarhizium anisopliae* | *Mahanarva fimbriolata, Zulia entreriana, Deois flavopicta* |
| 243 | 2022 | Andermatt | Spodovex; Littovir | *Spodoptera littoralis* nucleopolyhedrovirus | *Spodoptera frugiperda* |
| 244 | 2022 | Café Brasil | Mettaplus | *Metarhizium anisopliae* | *Mahanarva fimbriolata, Zulia entreriana, Deois flavopicta* |
| 245 | 2022 | Café Brasil | Bonvero | *Beauveria bassiana* | *Bemisia tabaci* biotype B*, Cosmopolites sordidus, Tetranychus urticae, Dalbulus maidis, Sphenophorus levis* |
| 246 | 2022 | Ballagro | Celtic | *Beauveria bassiana + Isaria javanica* | *Unaspis citri, Bemisia tabaci, Diaphorina citri* |
| 247 | 2022 | Hubio Biopar | Reporth | *Beauveria bassiana + Metarhizium anisopliae* | *Deois flavopicta, Euschistus heros* |
| 248 | 2022 | J. A. Moura Gonçalves | Biovéro; Beauveria MCP; Bravo BB; Bossbassi; BV Bassi; | *Beauveria bassiana* | *Bemisia tabaci* biotype B*, Cosmopolites sordidus, Tetranychus urticae, Dalbulus maidis, Sphenophorus levis* |
| 249 | 2022 | MB Enzymas | MBYO META; RNBIO META | *Metarhizium anisopliae* | *Mahanarva fimbriolata, Zulia entreriana, Deois flavopicta* |
| 250 | 2022 | MB Enzymas | MBYO BV; RNBIO BV | *Beauveria bassiana* | *Bemisia tabaci* biotype B*, Cosmopolites sordidus, Tetranychus urticae, Dalbulus maidis, Sphenophorus levis* |
| 251 | 2022 | Compostec Soluçôes | Compostec MCO Metha; movB2-Sc; | *Metarhizium anisopliae* | *Mahanarva fimbriolata, Zulia entreriana, Deois flavopicta* |
| 252 | 2022 | Compostec Soluçôes | Compostec MCO Betha; movB1-Sc; | *Beauveria bassiana* | *Bemisia tabaci* biotype B*, Cosmopolites sordidus, Tetranychus urticae, Dalbulus maidis, Sphenophorus levis* |
| 253 | 2022 | Massen | Metaleaf; Biogrun; Zebu; | *Metarhizium anisopliae* | *Mahanarva fimbriolata, Zulia entreriana, Deois flavopicta* |
| 254 | 2023 | Solubio | Lanceiro Bioagreen | *Beauveria bassiana + Metarhizium anisopliae* | *Deois flavopicta, Euschistus heros* |
| 255 | 2023 | J. A. Moura Gonçalves | Metatril; Metarhizium MCP; Mestra; Metra; MH ALL; | *Metarhizium anisopliae* | *Mahanarva fimbriolata, Zulia entreriana, Deois flavopicta* |
| 256 | 2023 | Uby | Gratto Bover | *Beauveria bassiana* | *Bemisia tabaci* biotype B*, Cosmopolites sordidus, Tetranychus urticae, Dalbulus maidis, Sphenophorus levis* |
| 257 | 2023 | Andermatt | Spodovir Plus | *Spodoptera littoralis* nucleopolyhedrovirus | *Spodoptera frugiperda* |
| 258 | 2023 | Bioma | Laphy Protection | *Spodoptera frugiperda* multiple nucleopolyhedrovirus | *Spodoptera frugiperda* |
| 259 | 2023 | Bio Insumos | Betk-03 | *Bacillus thuringiensis* | *Anticarsia gemmatalis* |
| 260 | 2023 | Koppert | Diplomata EVO | *Baculovirus Helicoverpa armigera + Chrysodeixis includens nucleopolyhedrovirus* | *Chrysodeixis includens, Helicoverpa armigera* |
| 261 | 2023 | Andermatt | Nomu-Protec | *Metarhizium rileyi* | *Spodoptera frugiperda* |
| 262 | 2023 | Solubio | Tec Catp 2.8; Comando BT-Bioagreen; | *Metarhizium anisopliae* | *Mahanarva fimbriolata, Zulia entreriana, Deois flavopicta* |
| 263 | 2023 | VSF | Caburé | *Metarhizium anisopliae* | *Mahanarva fimbriolata, Zulia entreriana, Deois flavopicta* |
| 264 | 2023 | Andermatt | BB-Protec, Beauvisan | *Beauveria bassiana* | *Bemisia tabaci* biotype B*, Cosmopolites sordidus, Tetranychus urticae, Dalbulus maidis, Sphenophorus levis* |
| 265 | 2023 | Base | Crisobase-E |  |  |
| 266 | 2023 | Elo | MBC Vetor; Eficaz Pro; |  |  |
| 267 | 2023 | Hubio Biopar | Soletra | *Beauveria bassiana* | *Bemisia tabaci* biotype B*, Cosmopolites sordidus, Tetranychus urticae, Dalbulus maidis, Sphenophorus levis* |
| 268 | 2023 | Uby | Gratto Meta | *Metarhizium anisopliae* | *Mahanarva fimbriolata, Zulia entreriana, Deois flavopicta* |
| 269 | 2023 | Bem da Terra | Mirah | *Beauveria bassiana* | *Bemisia tabaci* biotype B*, Cosmopolites sordidus, Tetranychus urticae, Dalbulus maidis, Sphenophorus levis* |
| 270 | 2023 | Bem da Terra | Titã | *Metarhizium anisopliae* | *Mahanarva fimbriolata, Zulia entreriana, Deois flavopicta* |
| 271 | 2023 | Agronatural | Bionature BM | *Beauveria bassiana + Metarhizium anisopliae* | *Deois flavopicta, Euschistus heros* |
| 272 | 2023 | Total Biotecnologia | BTP 066-20 SC | *Bacillus thuringiensis* | *Chrysodeixis includens, Spodoptera frugiperda, Tuta absoluta,Plutella xylostella, Helicoverpa armigera, Diatraea saccharalis, Ecdytolopha aurantiana* |
| 273 | 2023 | Bionat | Dissara; Bio Sirius; | *Metarhizium rileyi + Bacillus thuringienses* | *Helicoverpa armigera, Spodoptera eridania, Anticarsia gemmatalis, Spodoptera cosmioides, Spodoptera frugiperda* |
| 274 | 2023 | Vittia S.A. | Izaturbo; Isa-Vit; Isa-Vitt; Biozaria; | *Isaria javanica* | *Diaphorina citri, Bemisia tabaci* biotype B |
| 275 | 2023 | Total Biotecnologia | GRAPBeesECT | *Bacillus thuringiensis* | *Chrysodeixis includens, Spodoptera frugiperda, Plutella xylostella, Helicoverpa armigera, Ecdytolopha aurantian* |
| 276 | 2023 | Total Biotecnologia | BTP 0159-21 AC | *Bacillus thuringiensis; Brevibacillus laterosporus* | *Chrysodeixis includens, Spodoptera frugiperda, Tuta absoluta* |
| 277 | 2023 | Vittia S.A. | BV1219 | *Metarhizium anisopliae* | *Anthonomus grandis, Spodoptera frugiperda, Sphenophorus levis, Leucoptera coffeella* |
| 278 | 2023 | Hubio Biopar | Cryper | *Bacillus thuringiensis* var. kurstaki | *Alabama arqillacea, Spodoptera frugiperda, Chrysodeixis includens, Anticarsia gemmatalis* |
| 279 | 2023 | Agrobiológica Sustentabilidade S.A. | Metashock; Metatrix Evo; | *Metarhizium anisopliae* | *Deois flavopicta* |
| 280 | 2023 | Total Biotecnologia | Biobrev Full | *Bacillus thuringiensis + Brevibacillus laterosporus* | *Chrysodeixis includens, Spodoptera frugiperda, Helicoverpa armigera, Tuta absoluta, Diatraea saccharalis, Pseudaletia sequax* |
| 281 | 2023 | Total Biotecnologia | BTP 066-20A SC | *Bacillus thuringiensis* | *Chrysodeixis includens, Spodoptera frugiperda, Plutella xylostella, Helicoverpa armigera, Ecdytolopha aurantian, Tuta absoluta, Pseudaletia sequax, Diatraea saccharalis* |
| 282 | 2024 | Bionat | Spaiteran | *Beauveria bassiana* | *Sphenophorus levis, Hypothenemus hampei* |
| 283 | 2024 | Bionat | Orangel | *Beauveria bassiana* | *Diaphorina citri, Brevipalpus phoenics* |
| 284 | 2024 | Total Biotecnologia | BTP 0159-21AB | *Bacillus thuringiensis ; Brevibacillus laterosporus* | *Chrysodeixis includens, Spodoptera frugiperda, Plutella xylostella, Helicoverpa armigera, Ecdytolopha aurantian, Tuta absoluta, Pseudaletia sequax, Diatraea saccharalis* |
| 285 | 2024 | Viva | BovCombat Max | *Beauveria bassiana* | *Bemisia tabaci* biotype B*, Cosmopolites sordidus, Tetranychus urticae, Dalbulus maidis, Sphenophorus levis* |
| 286 | 2024 | Total Biotecnologia | BTP 068-20 SC-A | *Bacillus thuringiensis* | *Chrysodeixis includens, Spodoptera frugiperda, Plutella xylostella, Helicoverpa armigera, Ecdytolopha aurantian, Tuta absoluta, Pseudaletia sequax, Diatraea saccharalis* |
| 287 | 2024 | Biocross | Kelud | *Metarhizium anisopliae* | *Mahanarva fimbriolata, Zulia entreriana, Deois flavopicta* |
| 288 | 2024 | Biocross | Lassen | *Beauveria bassiana* | *Bemisia tabaci* biotype B*, Cosmopolites sordidus, Tetranychus urticae, Dalbulus maidis, Sphenophorus levis* |
| 289 | 2024 | Agrivalle | Auin | *Beauveria bassiana* | *Bemisia tabaci* biotype B*, Cosmopolites sordidus, Tetranychus urticae, Dalbulus maidis, Sphenophorus levis* |
| 290 | 2024 | Viva | Metacombat Pro | *Metarhizium anisopliae* | *Mahanarva fimbriolata, Zulia entreriana, Deois flavopicta* |
| 291 | 2024 | Biota | Menthar; Metaguard; Verdan; Questmeta; Metaguard; BS Protec; | *Metarhizium anisopliae* | *Mahanarva fimbriolata, Zulia entreriana, Deois flavopicta* |
| 292 | 2024 | Solefert | Metha Extreme | *Metarhizium anisopliae* | *Mahanarva fimbriolata, Zulia entreriana, Deois flavopicta* |
| 293 | 2024 | Massen | P&D 149 B | *Beauveria bassiana* | *Bemisia tabaci* biotype B*, Cosmopolites sordidus, Tetranychus urticae, Dalbulus maidis, Sphenophorus levis* |
| 294 | 2024 | Alfa | Evolut Duo | *Beauveria bassiana + Metarhizium anisopliae* | *Deois flavopicta, Euschistus heros* |
| 295 | 2024 | Organoplant | Fungus Hizium | *Metarhizium anisopliae* | *Mahanarva fimbriolata, Zulia entreriana, Deois flavopicta* |
| 296 | 2024 | J. A. Moura Gonçalves | Biometatril | *Beauveria bassiana + Metarhizium anisopliae* | *Deois flavopicta, Euschistus heros* |
| 297 | 2024 | Alisson Machado de Souza | Metabov; Atlasmix; Grifos | *Beauveria bassiana + Metarhizium anisopliae* | *Deois flavopicta, Euschistus heros* |
| 298 | 2024 | Alfa | Mavic Bio | *Metarhizium anisopliae* | *Mahanarva fimbriolata, Zulia entreriana, Deois flavopicta* |
| 299 | 2024 | Organoplant | Fungus Bveria; Fungus Bveria RBR; Fungus Bveria INC; Fungus Bveria MAX; Fungus Bveria OGN; Fungus Bveria GOLD | *Beauveria bassiana* | *Bemisia tabaci* biotype B*, Cosmopolites sordidus, Tetranychus urticae, Dalbulus maidis, Sphenophorus levis, Hypothenemus hampei* |
| 300 | 2024 | Simbiose | MethaProtection Evolution | *Metarhizium anisopliae* | *Scaptocoris castanea* |
| 301 | 2024 | Café Brasil | Mettabassi | *Beauveria bassiana + Metarhizium anisopliae* | *Deois flavopicta, Euschistus heros* |
| 302 | 2024 | VSF | Quero-Quero | *Beauveria bassiana + Metarhizium anisopliae* | *Deois flavopicta, Euschistus heros* |
| 303 | 2024 | Alfa | Aleris | *Beauveria bassiana* | *Bemisia tabaci* biotype B*, Cosmopolites sordidus, Tetranychus urticae, Dalbulus maidis, Sphenophorus levis, Hypothenemus hampei* |
| 304 | 2024 | Bioma | Vector Protection 2 | *Beauveria bassiana* | *Bemisia tabaci, Euschistus heros, Dichelops melacanthus, Dalbulus maidis* |
| 305 | 2024 | Noduagri | Noduveria L | *Beauveria bassiana* | *Bemisia tabaci* biotype B*, Cosmopolites sordidus, Tetranychus urticae, Dalbulus maidis, Sphenophorus levis, Hypothenemus hampei* |
| 306 | 2024 | Simbiose | Vir Protection H.A | *Baculovirus Helicoverpa armigera* | *Helicoverpa armigera* |
| 307 | 2024 | Biotrop | Bioturim | *Bacillus thuringiensis* | *Chrysodeixis includens, Spodoptera frugiperda, Pseudaletia sequax, Plutella xylostella, Helicoverpa armigera, Ecdytolopha aurantiana* |
| 308 | 2024 | FMC | Evedar | *Beauveria bassiana+ Metarhizium anisopliae* | *Tetranychus urticae, Frankliniella occidentalli, Hypothenemus hampei, Sphenophorus levis, Bemisia tabaci* biotype *B* |
| 309 | 2024 | Apoena | Forcety WP | *Metarhizium anisopliae* | *Mahanarva fimbriolata, Zulia entreriana, Deois flavopicta* |

**Table S2.** Overview of registered microbiological nematicide in Brazil.

| **N°** | **Year** | **Company** | **Product name** | **BCAs^1^** | **Targets** |
| --- | --- | --- | --- | --- | --- |
| 1 | 2007 | Koppert | Trichodermil SC 1306 | *Trichoderma harzianum* | *Pratylenchus brachyurus* |
| 2 | 2013 | Ballagro | Nemat; Nemaouro; | *Purpureocillium lilacinum* | *Meloidogyne javanica, Meloidogyne incognita,*  *Pratylenchus brachyurus* |
| 3 | 2016 | Stoller | Rizotec; Rizotec Crops; | *Pochonia chlamydosporia* | *Meloidogyne javanica* |
| 4 | 2016 | Simbiose | Nemacontrol | *Bacillus amyloliquefaciens* | *Pratylenchus brachyurus* |
| 5 | 2016 | Lallemand | Rizos OG | *Bacillus subtilis* | *Meloidogyne javanica,*  *Pratylenchus brachyurus* |
| 6 | 2016 | Lallemand | Onix OG | *Bacillus methylotrophicus* | *Meloidogyne javanica,*  *Pratylenchus brachyurus* |
| 7 | 2017 | FMC | Quartzo; Surface; | *Bacillus subtilis, Bacillus licheniformis* | *Meloidogyne exigua, Meloidogyne javanica, Meloidogyne incognita,*  *Pratylenchus brachyurus, Meloidogyne graminicola, Pratylenchus zeae,*  *Radopholus similis* |
| 8 | 2017 | FMC | Presence; Fortmax; | *Bacillus subtilis, Bacillus licheniformis* | *Meloidogyne javanica,*  *Pratylenchus brachyurus* |
| 9 | 2017 | Syngenta | Clariva PN | *Pasteuria nishizawae* | *Meloidogyne incognita, Pratylenchus brachyurus*  *Heterodera glycines* |
| 10 | 2017 | Koppert | Trianum WG | *Trichoderma harzianum* | *Pratylenchus brachyurus* |
| 11 | 2017 | Basf | Votivo Prime | *Bacillus firmus* | *Meloidogyne javanica,*  *Pratylenchus brachyurus* |
| 12 | 2017 | Basf | Oleaje Prime | *Bacillus firmus* | *Meloidogyne javanica,*  *Pratylenchus brachyurus* |
| 13 | 2017 | Basf | Andril Prime | *Bacillus firmus* | *Meloidogyne javanica,*  *Pratylenchus brachyurus* |
| 14 | 2018 | Lallemand | Rizos | *Bacillus subtilis* | *Meloidogyne javanica,*  *Pratylenchus brachyurus* |
| 15 | 2018 | Lallemand | Lalnix Over SC | *Bacillus methylotrophicus* | *Pratylenchus brachyurus* |
| 16 | 2018 | Syngenta | Clariva PN BR | *Pasteuria nishizawae* | *Meloidogyne incognita, Pratylenchus brachyurus*  *Heterodera glycines* |
| 17 | 2018 | Koppert | Daytona | *Trichoderma harzianum* | *Pratylenchus zeae* |
| 18 | 2018 | Lallemand | Lalnix Resist | *Trichoderma koningiopsis* | *Meloidogyne incognita, Pratylenchus brachyurus*  *Heterodera glycines* |
| 19 | 2018 | Agrobiológica | Nemakill | *Purpureocillium lilacinum* | *Meloidogyne incognita* |
| 20 | 2018 | Ballagro | Hope | *Paecilomyces lilacinus* | *Meloidogyne incognita* |
| 21 | 2018 | Koppert | Trianum DS | *Trichoderma harzianum* | *Pratylenchus brachyurus* |
| 22 | 2018 | Vittia | No-Nema; Nema-Attack; Nema-Guard; Bio Baciens; Cropwinner Eco Nemaxy; Vitalforce Bio Amilofaciens; Bionova Sanus S-Nema; Nematha; Klaatu; Nemavale; SKShiel; Glock Nema; | *Bacillus amyloliquefaciens* | *Meloidogyne incognita, Pratylenchus brachyurus,*  *Meloidogyne javanica,*  *Heterodera glycines* |
| 23 | 2018 | Vittia | Biobaci; Baci-Attack; Baci-Guard; Vitalforce Bio Phako; Cropwinner Eco Vastax; | *Bacillus subtilis* | *Meloidogyne incognita, Pratylenchus brachyurus,*  *Meloidogyne javanica,*  *Meloidogyne exigua,*  *Meloidogyne paranaenses,*  *Pratylenchus zeae* |
| 24 | 2019 | TZ Biotec | Purpureonyd FR 25 | *Purpureocillium lilacinum* | *Meloidogyne incognita* |
| 25 | 2019 | Ballagro | BN40.001/19 | *Purpureocillium lilacinum* | *Meloidogyne incognita* |
| 26 | 2019 | Agrivalle | Profix; Volga Goplan; | *Bacillus subtilis, Bacillus licheniformis, Purpureocillium lilacinum* | *Meloidogyne incognita, Pratylenchus brachyurus* |
| 27 | 2019 | Simbiose | Nemacontrol Super | *Bacillus amyloliquefaciens* | *Meloidogyne javanica, Pratylenchus brachyurus* |
| 28 | 2019 | Agrobiológica | Atialy; Lilatrix; | *Purpureocillium lilacinum* | *Meloidogyne incognita* |
| 29 | 2019 | Prophyto | Loyalty Bio; Trunemco; Vinemco; | *Bacillus amyloliquefaciens* | *Meloidogyne javanica,*  *Pratylenchus brachyurus, Meloidogyne incognita,*  *Rotylenchus reniformis* |
| 30 | 2020 | Koppert | Veraneio | *Bacillus amyloliquefaciens* | *Meloidogyne javanica,*  *Pratylenchus brachyurus, Meloidogyne incognita* |
| 31 | 2020 | Koppert | Boneville | *Bacillus amyloliquefaciens* | *Meloidogyne javanica,*  *Pratylenchus brachyurus, Meloidogyne incognita* |
| 32 | 2020 | Koppert | Chevelle | *Bacillus amyloliquefaciens* | *Meloidogyne javanica,*  *Pratylenchus brachyurus, Meloidogyne incognita* |
| 33 | 2020 | Sumitomo | Aveo EZ; Streat; | *Bacillus amyloliquefaciens* | *Pratylenchus brachyurus, Meloidogyne incognita*  *Heterodera glycines* |
| 34 | 2020 | Sumitomo | Lumialza | *Bacillus amyloliquefaciens* | *Pratylenchus brachyurus, Meloidogyne incognita*  *Heterodera glycines* |
| 35 | 2020 | Total Biotecnologia | Furatro; Nematrop; Biotrinsic Nemafree; Submax; | *Bacillus subtilis* | *Meloidogyne javanica,*  *Pratylenchus brachyurus, Meloidogyne incognita*  *Heterodera glycines* |
| 36 | 2020 | Total Biotecnologia | Nemaster | *Bacillus subtilis* | *Heterodera glycines*  *Meloidogyne javanica,*  *Pratylenchus brachyurus, Meloidogyne incognita* |
| 37 | 2020 | Total Biotecnologia | Paladyo | *Bacillus subtilis* | *Heterodera glycines*  *Meloidogyne javanica,*  *Pratylenchus brachyurus, Meloidogyne incognita* |
| 38 | 2020 | Agrivalle | Bio Tramo | *Bacilllus subtilis ; Bacillus licheniformis; Paecilomyces lilacinus* | *Pratylenchus zeae, Meloidogyne incognita,*  *Pratylenchus brachyurus* |
| 39 | 2020 | Massen | Messenger | *Bacillus subtilis, Bacillus licheniformis, Purpureocillium lilacinum* | *Meloidogyne incognita,*  *Pratylenchus brachyurus* |
| 40 | 2020 | Massen | AgDommon; Nemaoff; Bionexus; Volga; | *Bacillus subtilis, Bacillus licheniformis, Purpureocillium lilacinum* | *Meloidogyne incognita,*  *Pratylenchus brachyurus* |
| 41 | 2020 | Vittia | RIZO-TURBO; PC-Attack; PC-Guard; | *Pochonia chlamydosporia* | *Meloidogyne paranaenses, Meloidogyne incognita* |
| 42 | 2021 | Syngenta | Certano | *Bacillus velezensis* | *Meloidogyne incognita* |
| 43 | 2021 | Syngenta | Arvatico | *Bacillus velezensis* | *Meloidogyne incognita* |
| 44 | 2021 | Total Biotecnologia | Bioessence Drive | *Bacillus subtilis* | *Meloidogyne incognita,*  *Pratylenchus brachyurus,*  *Heterodera glycines,*  *Meloidogyne javanica* |
| 45 | 2021 | Mitsui & Co | Biostat WP; Gratto Nema; | *Purpureocillium lilacinum* | *Meloidogyne incognita,*  *Meloidogyne javanica* |
| 46 | 2021 | COMDEAGRO | AmyloTrop | *Bacillus amyloliquefaciens* | *Meloidogyne incognita,*  *Pratylenchus brachyurus,* |
| 47 | 2021 | Simbiose | Inlayon Eco | *Bacillus amyloliquefaciens* | *Meloidogyne javanica, Pratylenchus zeae, Pratylenchus brachyurus* |
| 48 | 2022 | Adama | Protege | *Bacillus amyloliquefaciens; Bacillus thuringiensis; Bacillus velezenis* | *Meloidogyne incognita,*  *Pratylenchus brachyurus,*  *Meloidogyne javanica,*  *Meloidogyne exigua,*  *Rotylenchus reniformis*  *Heterodera glycines* |
| 49 | 2022 | Total Biotecnologia | Biomagno; Bioharmer; | *Bacillus amyloliquefaciens; Bacillus thuringiensis; Bacillus velezenis* | *Meloidogyne incognita,*  *Pratylenchus brachyurus,*  *Meloidogyne javanica,*  *Meloidogyne exigua,*  *Rotylenchus reniformis* |
| 50 | 2022 | Total Biotecnologia | Quorum | *Bacillus amyloliquefaciens; Bacillus thuringiensis; Bacillus velezenis* | *Pratylenchus brachyurus* |
| 51 | 2022 | Koppert | KBR PDG07 | *Trichoderma harzianum* | *Meloidogyne incognita,*  *Pratylenchus brachyurus,*  *Heterodera glycines* |
| 52 | 2022 | CHR Hansen | Unnat | *Bacillus paralicheformis; Bacillus subtilis* | *Meloidogyne incognita,*  *Pratylenchus brachyurus,*  *Meloidogyne javanica,* |
| 53 | 2022 | CHR Hansen | Nimaxxa | *Bacillus paralicheformis; Bacillus subtilis* | *Meloidogyne incognita,*  *Pratylenchus brachyurus,*  *Meloidogyne exigua,*  *Rotylenchus reniformis,* *Pratylenchus zeae,*  *Heterodera glycines* |
| 54 | 2022 | Koppert | KBR PDG08 | *Trichoderma harzianum* | *Meloidogyne incognita,*  *Pratylenchus brachyurus,*  *Heterodera glycines* |
| 55 | 2022 | Bioma | Nema Protection | *Bacillus amyloliquefaciens* | *Pratylenchus brachyurus* |
| 56 | 2022 | Vittia | BV0518 | *Purporeocillium lilacinum* | *Meloidogyne incognita,*  *Heterodera glycines* |
| 57 | 2022 | Total Biotecnologia | BTP 007-19 | *Bacillus velezensis* | *Meloidogyne incognita* |
| 58 | 2022 | Andermatt | Nembac 42, Amyprotec 42 | *Bacillus velezensis* | *Meloidogyne incognita* |
| 59 | 2022 | Biota | Bamytis; Royalprezor; Basuty; Exclusiv; Batynem; Bany; Onprotect N; BRT Block; Bacill Mix; BS Aquanema; Bioboost; | *Bacillus amyloliquefaciens Bacillus subtilis; Bacillus thuringiensis* | *Meloidogyne incognita* |
| 60 | 2022 | COMDEAGRO | NemaZak | *Bacillus amyloliquefaciens Bacillus subtilis; Bacillus thuringiensis* | *Meloidogyne incognita* |
| 61 | 2023 | FMC | Presence Flex; Kalivar; Presence Full; | *Bacillus subtilis, Bacillus licheniformis* | *Meloidogyne exigua, Meloidogyne incognita,*  *Pratylenchus brachyurus, Meloidogyne graminicola, Pratylenchus zeae,*  *Radopholus similis, Heterodera glycines* |
| 62 | 2023 | Indigo | Biotrinsic N11 FP; Biotrinsic Habitans; Biotrinsic Nematrix; Indigo 407; | *Pseudomonas oryzihabitans* | *Meloidogyne incognita,*  *Pratylenchus brachyurus,*  *Heterodera glycines* |
| 63 | 2023 | Nooa | Auba(Artefato,Teros e Nerut) | *Bacillus amyloliquefaciens* | *Meloidogyne incognita* |
| 64 | 2023 | Solubio | SoluBio Raiz Performance; Raiz Protect; | *Bacillus subtilis* | *Meloidogyne incognita,*  *Pratylenchus brachyurus* |
| 65 | 2023 | Bioma | Nemax | *Bacillus amyloliquefaciens* | *Pratylenchus brachyurus* |
| 66 | 2023 | Bioma | Bioma - B.Bv 10 | *Bacillus velezensis* | *Meloidogyne incognita, Pratylenchus brachyurus* |
| 67 | 2023 | Bioma | Biagro Raiz | *Bacillus velezensis* | *Meloidogyne incognita, Pratylenchus brachyurus* |
| 68 | 2023 | Total Biotecnologia | Biolucro | *Bacillus licheniformis; Bacillus circulans;* *Paenibacillus azotofixans;* *Bacillus subtilis* | *Meloidogyne incognita,*  *Meloidogyne javanica* |
| 69 | 2023 | Solubio | Bio Solubilize; Phós Bio; | *Priestia megaterium* | *Pratylenchus zeae* |
| 70 | 2023 | Total Biotecnologia | BTP 177-21 | *Bacillus firmus* | *Meloidogyne incognita, Pratylenchus brachyurus* |
| 71 | 2023 | Andermatt | T-Protec | *Trichoderma asperellum* | *Heterodera glycines* |
| 72 | 2023 | Vital | Bionmt; Econema; | *Bacillus subtilis; Trichoderma harzianum* | *Meloidogyne incognita* |
| 73 | 2023 | Improcrop | RELI3VER | *Bacillus subtilis* | *Meloidogyne incognita* |
| 74 | 2023 | Total Biotecnologia | Pusher | *Bacillus subtilis* | *Meloidogyne javanica, Meloidogyne incognita, Pratylenchus brachyurus* |
| 75 | 2023 | Total Biotecnologia | BTP 500-21 | *Bacillus subtilis* | *Meloidogyne incognita* |
| 76 | 2023 | Solubio | DuoBalance Bioagreen; DuoBac-Bioagreen; SoluSafe; | *Bacillus amyloliquefaciens* | *Pratylenchus brachyurus* |
| 77 | 2024 | Bionat | Peregrino; Bio Pegasus; Canário; Glock Nema; | *Bacillus subtilis; Bacillus amyloliquefaciens* | *Meloidogyne javanica, Meloidogyne incognita, Pratylenchus brachyurus* |
| 78 | 2024 | Superbac | SuperShield | *Bacillus subtilis; Bacillus velezensis; Bacillus amyloliquefaciens; Bacillus licheniformis* | *Meloidogyne javanica, Meloidogyne incognita, Pratylenchus brachyurus* *Heterodera glycines; Pratylenchus zeae,* |
| 79 | 2024 | Ballagro | Nemat Stellus | *Purpureocillium lilacinum; Pochonia chlamydosporia* | *Meloidogyne javanica, Meloidogyne incognita, Pratylenchus brachyurus* *Heterodera glycines Rotylenchus reniformis* |
| 80 | 2024 | Ballagro | Voluto | *Paecilomyces lilacinus; Pochonia chlamydosporia* | *Pratylenchus brachyurus* |
| 81 | 2024 | Total Biotecnologia | BTP 002-18A | *Bacillus subtilis* | *Meloidogyne incognita, Pratylenchus brachyurus* |
| 82 | 2024 | Total Biotecnologia | BTP 500-21A | *Bacillus subtilis* | *Meloidogyne incognita* |
| 83 | 2024 | Innova | Offensive | *Purpureocillium lilacinum* | *Meloidogyne incognita* |
| 84 | 2024 | TZ Biotec | Pochonyd FR 25 | *Pochonia chlamydosporia* | *Meloidogyne incognita, Pratylenchus brachyurus* |
| 85 | 2024 | Prophyto | MBI-206 EP | *Burkholderia rinojensis* | *Pratylenchus brachyurus* |
| 86 | 2024 | Prophyto | Emenence TS | *Burkholderia rinojensis* | *Pratylenchus brachyurus* |
| 87 | 2024 | Prophyto | Rizonema | *Burkholderia rinojensis* | *Pratylenchus brachyurus* |
| 88 | 2024 | Total Biotecnologia | BTP 167-21A | *Paenibacillus azotofixans; Bacillus subtilis* | *Meloidogyne javanica, Meloidogyne incognita* |
| 89 | 2024 | Companhia Nitro Química | Gládios; Traex; | Paecilomyces lilacinus | *Meloidogyne incognita* |
| 90 | 2024 | Prophyto | Bafex | *Bacillus thuringiensis* | *Meloidogyne javanica* |
| 91 | 2024 | Solubio | Bio Gall | *Purpureocillium lilacinum* | *Meloidogyne incognita* |
| 92 | 2024 | FMC | Avizon | *Bacillus thuringiensis* | *Heterodera glycines, Rotylenchus reniformis* |
| 94 | 2024 | Solubio | Bio Poch | *Pochonia chlamydosporia* | *Meloidogyne javanica* |
| 95 | 2024 | Biotrop | Pusher | *Bacillus subtillis* | *Meloidogyne incógnita, Pratylenchus brachyurus, Pratylenchus zeae* |
| 96 | 2024 | Biotrop | BTP177-21A | *Bacillus firmus* | *Meloidogyne incógnita, Pratylenchus brachyurus* |

**Table S3.** Overview of registered microbiological fungicide in Brazil.

| **N°** | **Year** | **Company** | **Product name** | **BCAs^1^** | **Targets** |
| --- | --- | --- | --- | --- | --- |
| 1 | 2007 | Koppert | Trichodermil SC 1306 | *Trichoderma harzianum* | *Fusarium oxysporum, Rhizoctonia solani, Sclerotinia Sclerotiorum,*  *Thielaviopsis paradoxa* |
| 2 | 2011 | Bayer | Serenade | *Bacillus pumilus* | *Alternaria dauci, Alternaria porri, Botrytis cinérea, Colletotrichum acutatum,* *Colletotrichum gloeosporioides, Cryptosporiopsis perennans, Fusarium oxysporum, Mycosphaerella fijensis, Pythium ultimum, Sphaerotheca fuliginea, Sphaeroteca macularis,* *Streptomyces scabies, Xanthomonas citri subsp. Citri, Xanthomonas vesicatoria* |
| 3 | 2011 | Bayer | Sonata | *Bacillus amyloliquefaciens* | *Alternaria porri, Alternaria solani, Botrytis cinérea, Colletotrichum lindemuthianum, Cryptosporiopsis perennans, Sphaerotheca fuliginea, Sphaeroteca macularis, Uncinula necator* |
| 4 | 2011 | Lallemand | Quality | *Trichoderma asperellum* | *Fusarium oxysporum, Rhizoctonia solani, Sclerotinia Sclerotiorum* |
| 5 | 2011 | Novozymes | Trichodermax EC | *Trichoderma asperellum* | *Fusarium oxysporum, Rhizoctonia solani, Sclerotinia Sclerotiorum* |
| 6 | 2013 | Ballagro | Ecotrich WP; Tecnus; Dermavale; TrichoNext; Trichofull; Bionova Sanus Tricho; Grap Beestric; | *Trichoderma harzianum* | *Macrophomina phaseolina, Rhizoctonia solani, Sclerotinia Sclerotiorum* |
| 7 | 2015 | Ballagro | Predatox | *Trichoderma harzianum* | *Rhizoctonia solani, Sclerotinia Sclerotiorum* |
| 8 | 2016 | Lallemand | Organic WP | *Trichoderma harzianum* | *Fusarium oxysporum, Rhizoctonia solani* |
| 9 | 2016 | Simbiose. | Stimucontrol | *Trichoderma harzianum* | *Rhizoctonia solani, Sclerotinia Sclerotiorum* |
| 10 | 2016 | Iharabras | Eco-Shot | *Bacillus amyloliquefaciens* | *Alternaria dauci, Alternaria porri, Alternaria solani, Botrytis cinérea,*  *Colletotrichum gloeosporioides, Cryptosporiopsis perennans,*  *Erysiphe polygoni, Mycosphaerella fijiensis, Phyllosticta citricarpa,* *Sclerotinia sclerotiorum,* *Sphaerotheca fuliginea* |
| 11 | 2016 | UPL | Biobac; Tacap; | *Bacillus subtilis* | *Hemileia vastatrix, Neofabraea perennans, Rhizoctonia solani, Botrytis cinérea, Alternaria porri* |
| 12 | 2018 | Koppert | Daytona | *Trichoderma harzianum* | *Fusarium solani*, *Rhizoctonia solani, Sclerotinia Sclerotiorum,*  *Thielaviopsis paradoxa* |
| 13 | 2018 | Basf | Duravel | *Bacillus amyloliquefaciens* | *Botrytis cinerea Botrytis squamosa, Cryptosporiopsis perennans, Phyllosticta citricarpa, Pythium ultimum, Rhizoctonia solani, Sphaerotheca fuliginea, Streptomyces scabies,* *Uncinula necator Xanthomonas campestris Xanthomonas citri subsp. citri* |
| 14 | 2018 | Ballagro | Tritter | *Trichoderma harzianum* | *Rhizoctonia solani, Sclerotinia Sclerotiorum* |
| 15 | 2018 | Koppert | Walker | *Trichoderma harzianum* | *Fusarium oxysporum, Sclerotinia Sclerotiorum* |
| 16 | 2018 | Lallemand | Lalstop I32 SC | *Bacillus amyloliquefaciens* | *Botrytis cinerea* |
| 17 | 2018 | Agrivalle | Shocker, Peaky Goplan; Bio Venci; | *Bacillus amyloliquefaciens; Bacillus amyloliquefaciens; Trichoderma harzianum* | *Rhizoctonia solani, Sclerotinia Sclerotiorum, Fusarium solani* |
| 18 | 2018 | Vittia | Tricho-Turbo; Tricho-Attack; Tricho-Guard; VitalForce Bio Trico; CropWinner Eco Trico; Biologic Guard; Trichoking; Brandt SoloShield;SKProtect, Glock Evolution; Next Agro Easy Tharz; Tricosphera; | *Trichoderma asperellum* | *Rhizoctonia solani, Sclerotinia Sclerotiorum, Fusarium oxysporum, Macrophomina phaseolina* |
| 19 | 2018 | Vittia | No-Nema; Nema-Attack; Nema-Guard; Bio Baciens; Cropwinner Eco Nemaxy; Vitalforce Bio Amilofaciens; Bionova Sanus S-Nema; Nematha; Klaatu; Nemavale; SKShiel; Glock Nema; | *Bacillus amyloquefacens* | *Fusarium verticillioides, Macrophomina phaseolina* |
| 20 | 2018 | Vittia | Bio-imune; Multi-Attack; Multi-Guard; FungiOuro; Biologic Aerum; Brandt Clearfol; Power BAC Inductor;SKDefense; Solution Pro Evolution; | *Bacillus subtilis* | *Alternaria solani, Aspergillus Ochraceus, Botrytis cinérea,* *Colletotrichum acutatum,* *Colletotrichum gloeosporioides,*  *Colletotrichum lindemuthianum, Colletotrichum truncatum, Dechslera teres, Dechslera Tritici-repentis,* *Giberella zeae, Hemileia vastatrix, Neofabraea perennans, Phaeosphaeria maydis,* *Phakopsora pachyrhizi,* *Pseudomonas syringae, Pseudomonas syringae pv. garcae ,Puccinia triticina, Puccinia hordei,* *Ramularia aréola, Rhizoctonia solani, Sclerotinia sclerotiorum,* *Uncinula necator, Xanthomonas citri subsp. citri, Xanthomonas vesicatoria* |
| 21 | 2019 | TZ Biotec | Trichonyd FR 25 | *Trichoderma harzianum* | *Sclerotinia sclerotiorum* |
| 22 | 2019 | Mezfer | Natucontrol | *Trichoderma harzianum* | *Fusarium oxysporum, Rhizoctonia solani, Sclerotinia Sclerotiorum* |
| 23 | 2019 | Ballagro | Balin; Biotrinsic Trichoderma; | *Trichoderma harzianum* | *Rhizoctonia solani, Sclerotinia Sclerotiorum* |
| 24 | 2019 | Simbiose | Trich Protection | *Trichoderma harzianum* | *Rhizoctonia solani, Sclerotinia Sclerotiorum* |
| 25 | 2019 | Massen | Restrict; Zigurat; Vitanica Duo Protect; BioDoble; Amosbio; Wixx; Accuracy; |  |  |
| 26 | 2019 | Simbiose | Biagro Solo | *Trichoderma harzianum* | *Rhizoctonia solani, Sclerotinia Sclerotiorum* |
| 27 | 2020 | Ballagro | Ourotricx | *Trichoderma harzianum; Trichoderma asperellum; Bacillus amyloliquefaciens* | *Colletotrichum lindemuthianum, Macrophomina phaseolina, Rhizoctonia solani, Sclerotinia Sclerotiorum* |
| 28 | 2020 | Ballagro | Pardella; Primitivo; Ourotricx; Ourotricx; | *Trichoderma harzianum; Trichoderma asperellum; Bacillus amyloliquefaciens* | *Colletotrichum lindemuthianum, Macrophomina phaseolina, Rhizoctonia solani, Sclerotinia Sclerotiorum* |
| 29 | 2020 | Biota | Tanus | *Trichoderma harzianum; Trichoderma asperellum; Bacillus amyloliquefaciens* | *Colletotrichum lindemuthianum, Rhizoctonia solani, Sclerotinia Sclerotiorum* |
| 30 | 2020 | Simbiose | Stimucontrol Evolution | *Trichoderma harzianum* | *Sclerotinia Sclerotiorum* |
| 31 | 2020 | Total Biotecnologia | Furatro; Nematrop; Biotrinsic Nemafree; Submax; | *Bacillus subtilis* | *Rhizoctonia solani, Sclerotinia Sclerotiorum* |
| 32 | 2020 | Massen | Torpeno; Trichosmart; Bellator; Bionativus; Biofence; Praky; | *Bacillus amyloliquefaciens; Bacillus amyloliquefaciens; Trichoderma harzianum* | *Rhizoctonia solani, Sclerotinia Sclerotiorum* |
| 33 | 2020 | Agrivalle | Native | *Bacillus amyloliquefaciens; Bacillus amyloliquefaciens; Trichoderma harzianum* | *Rhizoctonia solani, Sclerotinia Sclerotiorum, Fusarium solani* |
| 34 | 2020 | Dillon | Bactel; DuoBac Meta; Easy Amylo; | *Bacillus amyloliquefaciens* | *Colletotrichum gloeosporioides,*  *Colletotrichum lindemuthianum* |
| 35 | 2020 | Massen | AgTecmmon | *Bacillus amyloliquefaciens; Bacillus amyloliquefaciens* | *Ascochyta coffeae, Cercospora coffeicola, Colletotrichum gloeosporioides, Colletotrichum lindemuthianum, Colletotrichum truncatum, Corynespora cassiicola,*  *Phaeosphaeria maydis,*  *Phoma costaricensis,*  *Ramularia areola* |
| 36 | 2020 | Agrivalle | Twixx-A; Bio Braza; Amosbio Goplan; | *Bacillus amyloliquefaciens; Bacillus amyloliquefaciens* | *Ascochyta coffeae, Cercospora coffeicola, Colletotrichum gloeosporioides, Colletotrichum lindemuthianum, Colletotrichum truncatum, Corynespora cassiicola, Phaeosphaeria maydis, Phoma costaricensis, Ramularia areola* |
| 37 | 2020 | Simbiose | Biagro Proteção | *Bacillus amyloliquefaciens* | *Colletotrichum gloeosporioides, Colletotrichum lindemuthianum* |
| 38 | 2020 | Genica | Congregga | *Trichoderma asperellum* | *Sclerotinea sclerotiorum* |
| 39 | 2020 | Agrivalle | Kamoi Pro; BioRoof; | *Clonostachys rosea* | *Botrytis cinerea* |
| 40 | 2020 | Bioma | Fx Protection | *Bacillus amyloliquefaciens* | *Colletotrichum gloeosporioides, Colletotrichum lindemuthianum* |
| 41 | 2021 | Syngenta | Certano | *Bacillus velezensis* | *Fusarium solani, Sclerotinia Sclerotiorum Rhizoctonia solani,* *Macrophomina phaseolina* |
| 42 | 2021 | Syngenta | Arvatico | *Bacillus velezensis* | *Fusarium solani, Rhizoctonia solani,* *Macrophomina phaseolina* |
| 43 | 2021 | Koppert | BTP 005-19 | *Bacillus pumilus* | *Cercospora kikuchii, Cercospora kikuchii, Septoria glycines* |
| 44 | 2021 | Koppert | Caravan | *Bacillus pumilus* | *Cercospora kikuchii, Cercospora kikuchii, Septoria glycines* |
| 45 | 2021 | COMDEAGRO | Amitrix SC | *Bacillus amyloliquefaciens* | *Colletotrichum gloeosporioides, Colletotrichum lindemuthianum* |
| 46 | 2021 | FMC | Ataplan; Aratel; | Bacillus velezensis; Bacillus subtilis | *Pythium aphanidermatum, Pythium ultimum, Rhizoctonia solani, Fusarium oxysporum, Colletotrichum truncatum* |
| 47 | 2021 | Agrobiológica | Tríppel; Trichotrix; | *Trichoderma asperellum* | *Fusarium oxysporum, Rhizoctonia solani, Sclerotinia Sclerotiorum* |
| 48 | 2021 | Companhia Nitro Química | Trichodermaiz WP; Garantte; Trichodermaiz Plus WP; Trichodermaiz WP PRO; | *Trichoderma harzianum* | *Sclerotinia Sclerotiorum* |
| 49 | 2021 | Mezfer | Baktillis | *Bacillus subtilis* | *Colletotrichum gloeosporioides, Fusarium solani, Rhizoctonia solani,* |
| 50 | 2021 | Biota | Velez; Questmyll; Onceres; BRT Protect; Gardeon; BS Inox; Ajaax; | *Bacillus amyloliquefaciens* | *Colletotrichum gloeosporioides,*  *Colletotrichum lindemuthianum* |
| 51 | 2021 | Simbiose | GreenControl | *Trichoderma harzianum* | *Fusarium solani, Sclerotinia Sclerotiorum* |
| 52 | 2021 | Agrobiológica | Amanzi | *Bacillus amyloliquefaciens* | *Colletotrichum gloeosporioides,*  *Colletotrichum lindemuthianum* |
| 53 | 2021 | COMDEAGRO | Tricozak | *Trichoderma harzianum; Trichoderma asperellum; Bacillus amyloliquefaciens* | *Colletotrichum lindemuthianum, Rhizoctonia solani, Sclerotinia Sclerotiorum* |
| 54 | 2021 | Mitsui & Co | Amylo-X SL; Bacilo-X; | *Bacillus amyloliquefaciens* | *Alternaria porri, Rhizoctonia solani, Sphaerotheca fuliginea, Sclerotinea sclerotiorum, Colletotrichum gloeosporioides, Botrytis cinérea, Pythium ultimum, Mycosphaerella fijensis* |
| 55 | 2021 | Total Biotecnologia | Reverb | *Bacillus velezensis; Bacillus subtilis; Bacillus pumilus* | *Cercospora kikuchii Cercospora zeae-maydis, Cercosporidium personatum, Colletotrichum acutatum, Colletotrichum lindemuthianum, Colletotrichum truncatum, Corynespora cassiicola, Exserohilum turcicum, Hemileia vastatrix, Puccinia triticina, Ramularia aréola, Sclerotinia sclerotiorum, Septoria glycines, Sphaerotheca fuliginea, Stenocarpella maydis* |
| 56 | 2021 | Total Biotecnologia | Bombardeiro; Lastro; | *Bacillus subtilis; Bacillus velezensis; Bacillus pumilus* | *Colletotrichum falcatum, Cercospora kikuchii Cercospora zeae-maydis, Cercosporidium personatum, Colletotrichum acutatum, Colletotrichum truncatum, Corynespora cassiicola, Exserohilum turcicum, Hemileia vastatrix, Puccinia triticina, Sclerotinia sclerotiorum, Septoria glycines, Sphaerotheca fuliginea, Stenocarpella maydis* |
| 57 | 2021 | Rizobacter | Rizoderma TSI; Rizoderma TSI Bio Fungicida; Rizoderma; | *Trichoderma afroharzianum* | *Fusarium oxysporum, Rhizoctonia solani, Sclerotinia Sclerotiorum* |
| 58 | 2021 | Genica | Gladiador | *Trichoderma asperellum* | *Sclerotinea sclerotiorum* |
| 59 | 2021 | Agrobiológica | Powerfung | *Trichoderma asperellum* | *Fusarium oxysporum, Rhizoctonia solani, Sclerotinia Sclerotiorum* |
| 60 | 2022 | FMC | Provilar | *Bacillus velezensis; Bacillus subtilis* | *Sclerotinea sclerotiorum, Corynespora cassiicola* |
| 61 | 2022 | Lallemand | Lalstop Organic DS | *Trichoderma asperellum* | *Fusarium solani, Rhizoctonia solani* |
| 62 | 2022 | Adama | Protege | *Bacillus amyloliquefaciens ;Bacillus thuringiensis; Bacillus velezensis* | *Ceratocystis paradoxa, Rhizoctonia solani, Macrophomina phaseolina* |
| 63 | 2022 | Symborg | Thydra | *Trichoderma harzianum* | *Colletotrichum lindemuthianum, Sclerotinea sclerotiorum* |
| 64 | 2022 | Total Biotecnologia | Biomagno; Bioharmer; | *Bacillus amyloliquefaciens; Bacillus thuringiensis; Bacillus velezensis* | *Ceratocystis paradoxa, Macrophomina phaseolina, Rhizoctonia solani* |
| 65 | 2022 | Biomip | Biotricho | *Trichoderma harzianum; Trichoderma viride* | *Fusarium solani, Rhizoctonia solani* |
| 66 | 2022 | Genica | GNC 006-3 | *Trichoderma asperellum* | *Sclerotinea sclerotiorum* |
| 67 | 2022 | Total Biotecnologia | Bioshield | *Bacillus subtilis; Bacillus velezensis; Bacillus pumilus* | *Cercospora kikuchii Cercospora zeae-maydis, Cercosporidium personatum, Colletotrichum acutatum, Colletotrichum lindemuthianum, Colletotrichum truncatum, Corynespora cassiicola, Exserohilum turcicum, Hemileia vastatrix, Puccinia triticina, Ramularia aréola, Sclerotinia sclerotiorum, Septoria glycines, Sphaerotheca fuliginea, Stenocarpella maydis* |
| 68 | 2022 | Total Biotecnologia | Quorum | *Bacillus amyloliquefaciens; Bacillus thuringiensis; Bacillus velezensis* | *Rhizoctonia solani* |
| 69 | 2022 | Koppert | KBR PDG07 | *Trichoderma harzianum* | *Sclerotinia Sclerotiorum, Macrophomina phaseolina Rhizoctonia solani, Fusarium solani* |
| 70 | 2022 | Koppert | KBR PDG08 | *Trichoderma harzianum* | *Macrophomina phaseolina Rhizoctonia solani, Fusarium solani* |
| 71 | 2022 | Bionat | Habitat | *Trichoderma afroharzianum* | *Rhizoctonia solani, Sclerotinia Sclerotiorum* |
| 72 | 2022 | Bionat | Bioatria | *Trichoderma afroharzianum* | *Rhizoctonia solani, Sclerotinia Sclerotiorum* |
| 73 | 2022 | JCO | Trichoagro JCO | *Trichoderma harzianum; Trichoderma viride* | *Fusarium oxysporum, Rhizoctonia solani* |
| 74 | 2022 | Solubio | TrikoSoil | *Trichoderma harzianum; Trichoderma viride* | *Fusarium oxysporum, Rhizoctonia solani* |
| 75 | 2022 | Solubio | Trincheira Bioagreen; Trincheira-Bioagreen; Trikofit; Triko Bio; | *Trichoderma harzianum; Trichoderma asperellum; Bacillus amyloliquefaciens* | *Sclerotinia Sclerotiorum* |
| 76 | 2022 | Solubio | Marechal BioGreen; Marechal-Bioagreen; Trichomais PM; Green Bio; | *Trichoderma harzianum* | *Sclerotinia Sclerotiorum* |
| 77 | 2022 | TZ Biotec | Trichoningyd FR 25 | *Trichoderma koningiopsis* | *Fusarium oxysporum* |
| 78 | 2022 | Total Biotecnologia | BTP 007-19 | *Bacillus velezensis* | *Fusarium oxysporum* |
| 79 | 2022 | Biossíntese | Trichofourt | *Trichoderma harzianum; Trichoderma viride* | *Fusarium oxysporum, Rhizoctonia solani* |
| 80 | 2022 | TZ Biotec | Trichonyd RS FR 25 | *Trichoderma reesei* | *Fusarium oxysporum* |
| 81 | 2022 | Bom Futuro | Trichoderma Bom Futuro | *Trichoderma harzianum; Trichoderma viride* | *Fusarium oxysporum, Rhizoctonia solani* |
| 82 | 2022 | Café Brasil | Trichoadvance | *Trichoderma harzianum* | *Sclerotinia Sclerotiorum* |
| 83 | 2022 | MB Enzymas | MBYO TRIC; RNBIO TRIC | *Trichoderma harzianum; Trichoderma viride* | *Fusarium solani,, Rhizoctonia solani* |
| 84 | 2023 | Solatus | Tivra | *Trichoderma harzianum; Trichoderma viride* | *Fusarium oxysporum, Rhizoctonia solani* |
| 85 | 2023 | Hubio Biopar | Rethax | *Trichoderma harzianum,; Trichoderma viride* | *Fusarium oxysporum, Rhizoctonia solani* |
| 86 | 2023 | Promip | Harzimip; Glock Evolution WP; | *Trichoderma harzianum* | *Sclerotinia Sclerotiorum* |
| 87 | 2023 | Oligos | Oligos 4 T | *Trichoderma harzianum; Trichoderma viride* | *Fusarium oxysporum, Rhizoctonia solani* |
| 88 | 2023 | Andermatt | Atroverde 77; T-77; | *Thichoderma atroviride* | *Sclerotinia Sclerotiorum* |
| 89 | 2023 | Elo Simbiotica | Nem Phoco | *Trichoderma harzianum; Trichoderma viride* | *Fusarium oxysporum, Rhizoctonia solani* |
| 90 | 2023 | Agronatural | Bionature Tricho | *Trichoderma harzianum,; Trichoderma viride* | *Fusarium oxysporum, Rhizoctonia solani* |
| 91 | 2023 | HB | Trichobach | *Trichoderma harzianum,; Trichoderma viride* | *Fusarium oxysporum, Rhizoctonia solani* |
| 92 | 2023 | Syngenta | Taegro e Krivesta | *Bacillus amyloliquefaciens* | *Alternaria solani,* *Uncinula necator Rhizoctonia solani, Sphaerotheca fuliginea, Sclerotinea sclerotiorum, Colletotrichum gloeosporioides, Botrytis cinerea* |
| 93 | 2023 | Tradecorp | Row-Vispo | *Bacillus subtilis* | *Phakopsora pachyrhizi, Hemileia vastatrix* |
| 94 | 2023 | Genica | GNC 006-4 | *Trichoderma asperellum* | *Sclerotinea sclerotiorum* |
| 95 | 2023 | Total Biotecnologia | BTP 173-21A | *Trichoderma harzianum; Trichoderma viride* | *Fusarium oxysporum, Rhizoctonia solani* |
| 96 | 2023 | Simbiose | FrontierControl | *Bacillus velenzis* | *Pantoe Ananatis, Sclerotinea sclerotiorum* |
| 97 | 2023 | Total Biotecnologia | BTP 173-21 | *Trichoderma harzianum; Trichoderma viride* | *Fusarium oxysporum, Rhizoctonia solani* |
| 98 | 2023 | Tradecorp | Milarum | *Bacillus subtilis* | *Phytophthora infestans, Pseudoperonospora cubensis, Fusarium solani, Rhizoctonia solani* |
| 99 | 2023 | Bio Insumos | Nacillus Max | *Bacillus Licheniformis; Bacillus subtilis; Brevibacillus parabrevis* | *Alternaria solani, Fusarium solani, Rhizoctonia solani* |
| 100 | 2023 | Toyobo | Biotrix; Agtrimmon; Neotri Power; | *Trichoderma harzianum* | *Sclerotinea sclerotiorum* |
| 101 | 2023 | Tradecorp | Ospo Vi55 | *Bacillus subtilis* | *Leveillula taurica,*  *Sphaerotheca fuliginea*  *Uncinula necator* |
| 102 | 2023 | Total Biotecnologia | Biolucro | *Bacillus circulans; Paenibacillus azotofixans; Bacillus subtilis; Bacillus licheniformis* | *Rhizoctonia solani, Macrophomina phaseolina* |
| 103 | 2023 | Biota | Tryba | *Trichoderma harzianum; Trichoderma asperellum; Bacillus amyloliquefaciens; Bacillus subtilis* | *Rhizoctonia solani* |
| 104 | 2023 | Bem da Terra | Garantte; Trichocombat Pro; Ontrich; | *Trichoderma harzianum* | *Sclerotinea sclerotiorum* |
| 105 | 2023 | Biota | Harz WP; BS Tricho Duo; Ontrich;Trichoboost; Enhancer; | *Trichoderma harzianum* | *Sclerotinea sclerotiorum* |
| 106 | 2023 | Uby | Gratto Harz | *Trichoderma harzianum* | *Sclerotinea sclerotiorum* |
| 107 | 2023 | Sinon | Sinsmart | *Bacillus amyloliquefaciens* | *Xanthomonas campestres, Sphaerotheca fuliginea, Rhizoctonia solani, Pythium ultimum, Fusarium solani, Botrytis cinerea* |
| 108 | 2023 | Ecosolução | T-Rex, Hungry, Unity, Grenade, Angry, Vingador e Revenge | *Trichoderma harzianum; Trichoderma viride* | *Fusarium oxysporum, Rhizoctonia solani* |
| 109 | 2023 | Solubio | Bio Release | *Pseudomonas fluorescens* | *Rhizoctonia solani* |
| 110 | 2024 | Superbac | Superguard | *Bacillus subtillis; Bacilus amyloliquefaciens* | *Fusarium oxysporum, Rhizoctonia solani; Sclerotinea sclerotiorum* |
| 111 | 2024 | Indigo | Biotrinsic D451 FP; Biotrinsic Hamatum; Biotrinsic Fitocontrol; Indigo 451; Indigo Hamatum | *Trichoderma hamatum* | *Fusarium solani, Fusarium verticillioides, Macrophomina phaseolina, Phytophthora sojae, Rhizoctonia solani, Sclerotinia sclerotiorum, Stenocarpella maydis* |
| 112 | 2024 | Simbiose | Stimu Protection | *Trichoderma harzianum* | *Rhizoctonia solani, Sclerotinia Sclerotiorum, Colletotrichum lindemuthianum* |
| 113 | 2024 | VSF | Canindé | *Trichoderma harzianum* | *Sclerotinea sclerotiorum* |
| 114 | 2024 | JCO | Trichotaurus JCO | *Trichoderma harzianum* | *Sclerotinea sclerotiorum* |
| 115 | 2024 | Total Biotecnologia | BTP 167-21A | *Bacillus circulans; Paenibacillus azotofixans; Bacillus subtilis; Bacillus licheniformis* | *Macrophomina phaseolina, Rhizoctonia solani* |
| 116 | 2024 | J. A. Moura Gonçalves | Tricho-Harmax | *Trichoderma harzianum* | *Sclerotinea sclerotiorum* |
| 117 | 2024 | Companhia Nitro Química | Égide | *Bacillus amyloliquefaciens; Bacillus velezensis* | *Botrytis cinerea* |
| 118 | 2024 | Noduagri | Noduderma | *Trichoderma harzianum* | *Sclerotinea sclerotiorum* |
| 119 | 2024 | Viva | Trichombat | *Trichoderma harzianum* | *Sclerotinea sclerotiorum* |
